# N6-Isopentenyladenosine Inhibits Colorectal Cancer and Improves Sensitivity to 5-Fluorouracil Targeting FBXW7 Tumor Suppressor

**DOI:** 10.3390/cancers11101456

**Published:** 2019-09-28

**Authors:** Donatella Fiore, Chiara Piscopo, Maria Chiara Proto, Michele Vasaturo, Fabrizio Dal Piaz, Bruno Marcello Fusco, Cristina Pagano, Chiara Laezza, Maurizio Bifulco, Patrizia Gazzerro

**Affiliations:** 1 Department of Pharmacy, University of Salerno, 84084 Fisciano (SA), Italy; dfiore@unisa.it (D.F.); cpiscopo@unisa.it (C.P.); maproto@unisa.it (M.C.P.); mvasaturo@unisa.it (M.V.); fuscobr@unisa.it (B.M.F.); 2 PhD Program in Drug Discovery and Development, University of Salerno, Via Giovanni Paolo II 132, 84084 Fisciano (SA), Italy; 3 Department of Medicine, Surgery and Dentistry, University of Salerno, 84081 Baronissi (Salerno), Italy; fdalpiaz@unisa.it; 4 Department of Molecular Medicine and Medical Biotechnologies, University of Naples “Federico II”, 80131 Naples, Italy; pagano.cris@gmail.com (C.P.); chilaez@hotmail.com (C.L.); maubiful@unina.it (M.B.); 5 Institute of Endocrinology and Experimental Oncology “Gaetano Salvatore” (IEOS), National Research Council (CNR), 80131 Naples, Italy

**Keywords:** N6-isopentenyladenosine, colon cancer, FBXW7, mevalonate pathway, 5-fluorouracil, chemoresistance

## Abstract

N6-isopentenyladenosine has been shown to exert potent in vitro antitumor activity on different human cancers, including colorectal cancer. Although some potential biochemical targets have been identified, its precise mechanism of action remains unclear. We found that N6-isopentenyladenosine affects colorectal cancer proliferation in in vitro models carrying different mutational status of *FBXW7* and *TP53* genes, and in HCT116 xenografts in SCID mice, by increasing the expression of the well-established tumor suppressor FBXW7, a component of the SCF-E3 ubiquitin ligase complex that promotes degradation of various oncoproteins and transcription factors, such as c-Myc, SREBP and Mcl1. Corroborating our previous studies, we identified for the first time the FBXW7/SREBP/FDPS axis as a target of the compound. Pull down of ubiquitinated proteins, immunoprecipitation and luciferase assays, reveal that through the increase of FBXW7/c-Myc binding, N6-isopentenyladenosine induces the ubiquitination of c-Myc, inhibiting its transcriptional activity. Moreover, in *FBXW7*- and *TP53*-wild type cells, N6-isopentenyladenosine strongly synergizes with 5-Fluorouracil to inhibit colon cancer growth in vitro. Our results provide novel insights into the molecular mechanism of N6-isopentenyladenosine, revealing its multi-targeting antitumor action, in vitro and in vivo. Restoring of FBXW7 tumor-suppressor represents a valid therapeutic tool, enabling N6-isopentenyladenosine as optimizable compound for patient-personalized therapies in colorectal cancer.

## 1. Introduction

N6-isopentenyladenosine (IPA) is a member of cytokinin family [1], found to exert antitumor activity on a wide range of human epithelial cancer cells [2], including colorectal cancer (CRC) [3,4]. Although it has been largely accepted that IPA inhibits farnesyl diphosphate synthase (FDPS) enzyme [5,6], the exact molecular mechanism underlying the antitumor efficacy is still unknown.

The modified nucleoside’s chemical structure allows IPA to potentially influence multiple pathways, explaining its pleiotropic action. It was previously reported that IPA exerts antiangiogenic effect, through activation of AMP-Kinase (AMPK) that allows mTOR inhibition, autophagosome accumulation and late-stage autophagy inhibition. This effect seems to comply with the inhibition of Rab7 prenylation, as expected of IPA ability to inhibit FDPS [7,8].

Interestingly, IPA showed also immune-modulating properties activating NK cells-mediated recognition of glioma cells, achieved by the upregulation of ULBP2, NKG2D ligand, in *TP53* wild type cells or MICA/B in p53 mutant gliomas [9]. Among the identified molecular mechanisms, in glioblastoma multiforme (GBM), IPA induces c-Cbl-mediated EGFR ubiquitination, inhibiting its downstream signaling [10].

Gene expression profile in breast and lung cancer cells highlighted a significant upregulation of genes encoding for proteins (or domains of them) strongly involved in ubiquitin proteasome system (UPS) regulation. Among these, the upregulation of WD Repeat and SOCS box-containing 1 (*WSB1*), F-box and leucine-rich repeat protein 12 (*FBXL12*) and Ras-like without CAAX 1 (*RIT1*, also known as *RBX1*) genes [11] seems to be a remarkable evidence, since F-box proteins and SCF ligases, received gaining attention as molecular targets in several malignancies, including CRC [12].

Among the hundreds of E3 ligases already characterized, SCF^FBXW7^ is one of the best studied in CRC. It is clear that FBXW7 (F-box and WD repeat domain-containing 7) plays the role of tumor suppressor, being involved in ubiquitination and degradation of several oncoproteins, such as c-Myc, Cyclin E, Notch, SREBP (sterol regulatory element-binding protein), Mcl1 and many others [13,14,15,16]. In CRC tissues, low expression of FBXW7 mRNA correlates with the stage of disease progression and poor prognosis. Moreover, silencing or inactivating mutations of *FBXW7* cause hyper-proliferation of CRC cells, due to an aberrant expression and activity of c-Myc and Cyclin E [17]. Several authors demonstrated the tumor suppressive role of RNF20 (ring finger protein 20) E3 ligase that, together with H2B monoubiquitination (H2BUb), also modulates colon cancer-associated inflammation [18,19]. Furthermore, in CRC patients, H2BUb seems to be inversely correlated to malignant progression [20].

In the present work, we provide novel clues about IPA molecular mechanism. We found that, through upregulation of FBXW7, IPA inactivates c-Myc at transcriptional level and affects FBXW7/SREBP/FDPS axis, enforcing previous published findings. Last but not least, the in vivo efficacy and the finding that IPA ameliorates in vitro 5-fluorouracil (5-FU) activity in CRC, make IPA an intriguing lead compound for antitumor therapies.

## 2. Results

### 2.1. N6-Isopentenyladenosine Triggers Cell Death in CRC Cells, In Vitro

**Figure 1:**
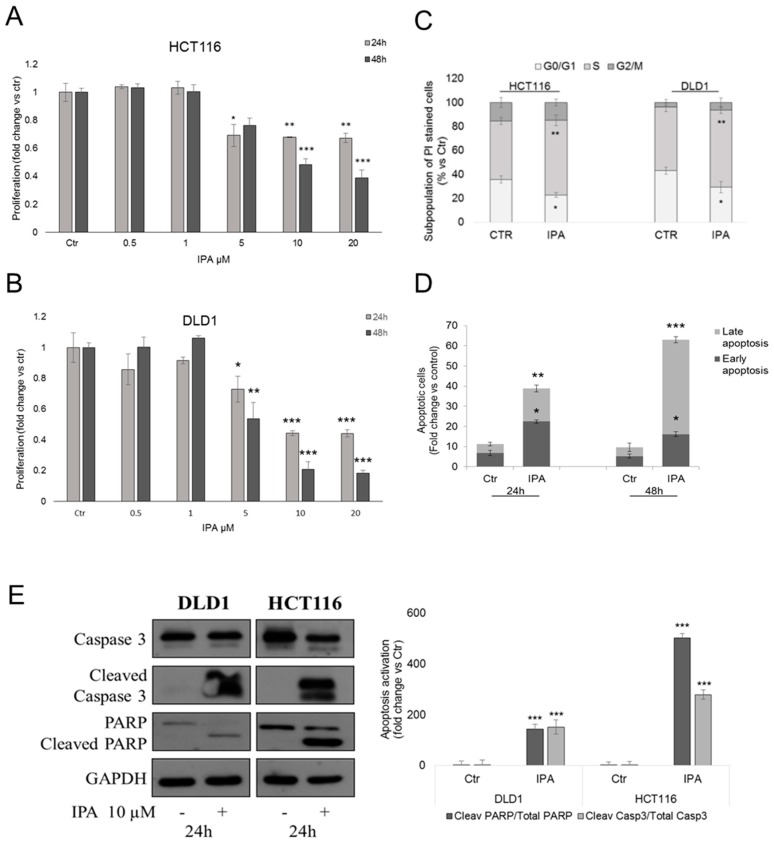
Effect of IPA on CRC cells in vitro. BrdU incorporation assay in HCT116 (**A**) and DLD1 (**B**) cells treated with indicated concentrations of IPA, for 24 h or 48 h. (**C**) Cell cycle analysis of HCT116 and DLD1 cells treated with IPA for 24 h and stained with PI. (**D**) Cytofluorimetric apoptosis analysis of Annexin V-FITC/PI double-stained DLD1 cells treated with 10 µM IPA, for 24 h and 48 h. Data are expressed as mean ± standard deviation (SD) of five independent experiments in triplicate. * *p* < 0.05, ** *p* < 0.01, *** *p* < 0.005 versus (vs.) control. (**E**) Representative western blot and densitometry analysis of apoptotic pathway activation in DLD1 and HCT116 cells. GAPDH was used as loading control. Data are expressed as mean ± SD of three independent experiments. *** *p* < 0.005 vs. control.

Previous findings evidenced the in vitro antiproliferative effect of IPA in human cancers [3,4]. Here we reported that IPA significantly inhibited DLD1 and HCT116 CRC cells proliferation, in a dose- and time-dependent manner, starting from 5 µM after 24 h of treatment (Figure 1A,B). Cytofluorimetric analysis of PI stained DLD1 and HCT116 cells, revealed that the treatment with IPA arrested the cells in the S phase of cell cycle (Figure 1C). To understand if inhibition of proliferation and S phase block matched with cell death induction, we performed apoptosis assay. Accordingly with previous results, IPA significantly increased PI/Annexin V-FITC double stained cells, suggesting the induction of apoptotic death (Figure 1D). Apoptosis was further confirmed by western blot analysis of activated Caspase 3 and PARP (Figure 1E), both cleaved after 24 h of treatment.

### 2.2. IPA Upregulates FBXW7 Expression

**Figure 2:**
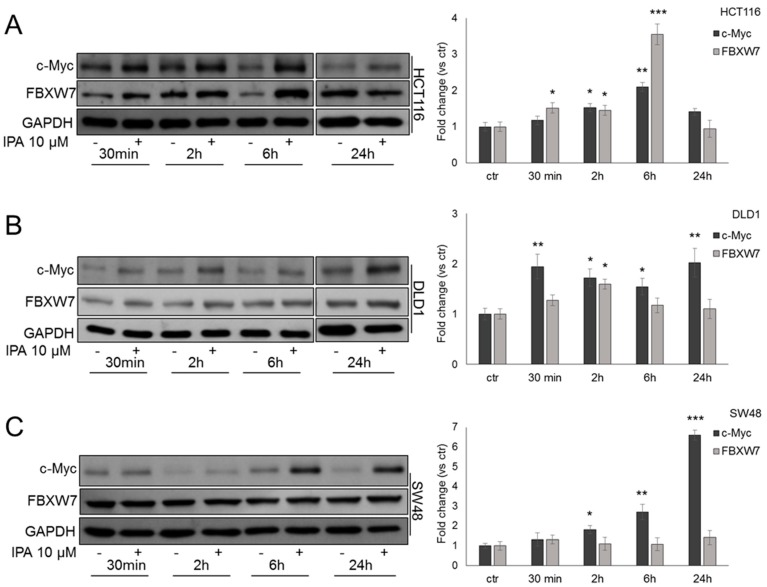
IPA modulates FBXW7 and c-Myc expression. Representative western blot and relative densitometry analysis (histograms on the right) of FBXW7 and c-Myc protein expression in HCT116 (**A**), DLD1 (**B**) and SW48 (**C**) cells, treated with IPA at the indicated time points. Blots are representative of at least five independent experiments. Data are expressed as mean ± SD of five independent experiments. * *p* < 0.05, ** *p* < 0.01, *** *p* < 0.005 vs. control.

Our and others’ previous studies suggested that IPA seems to be involved in UPS regulation [10,11]. We then focused our attention on FBXW7, tumor-suppressor strongly implicated in colon carcinogenesis. Since c-Myc is a well-known substrate of FBXW7 [14], we analyzed its protein expression in DLD1 and HCT116 cells harboring wild type *FBXW7* gene [21], starting from 30 min of treatment with IPA. In HCT116 cells, IPA induced c-Myc expression after 2 h and 6 h of treatment. Coherently, FBXW7 expression was strongly induced by IPA after 6 h of treatment, but after 24 h of IPA exposure, compared to untreated cells, the levels of both c-Myc and FBXW7 tended to decrease (Figure 2A). In DLD1 cells—that have a high percentage of methylated CpG sites in *FBXW7* promoter [22]—expression trend after treatment with IPA was slightly different compared to HCT116. c-Myc expression showed a biphasic expression while FBXW7 expression increased after 2 h of treatment, when c-Myc levels tended to decrease (Figure 2B).

To gain further evidence about the involvement of FBXW7-c-Myc crosstalk in IPA molecular mechanism, we analyzed its effect in SW48 cells, harboring *FBXW7* heterozygous frame-shift deletion (c.2001delG) that affects substrate binding [21]. First, we observed that—as in DLD1 and HCT116 cells—IPA significantly inhibited SW48 proliferation and induced apoptosis (Supplementary Figure S1). As expected, compared to untreated cells, IPA was not able to induce FBXW7 expression leading to a time-dependent accumulation of c-Myc (Figure 2C), most likely due to the loss of FBXW7-dependent degradation.

### 2.3. IPA Inhibits c-Myc Transcriptional Activity

**Figure 3:**
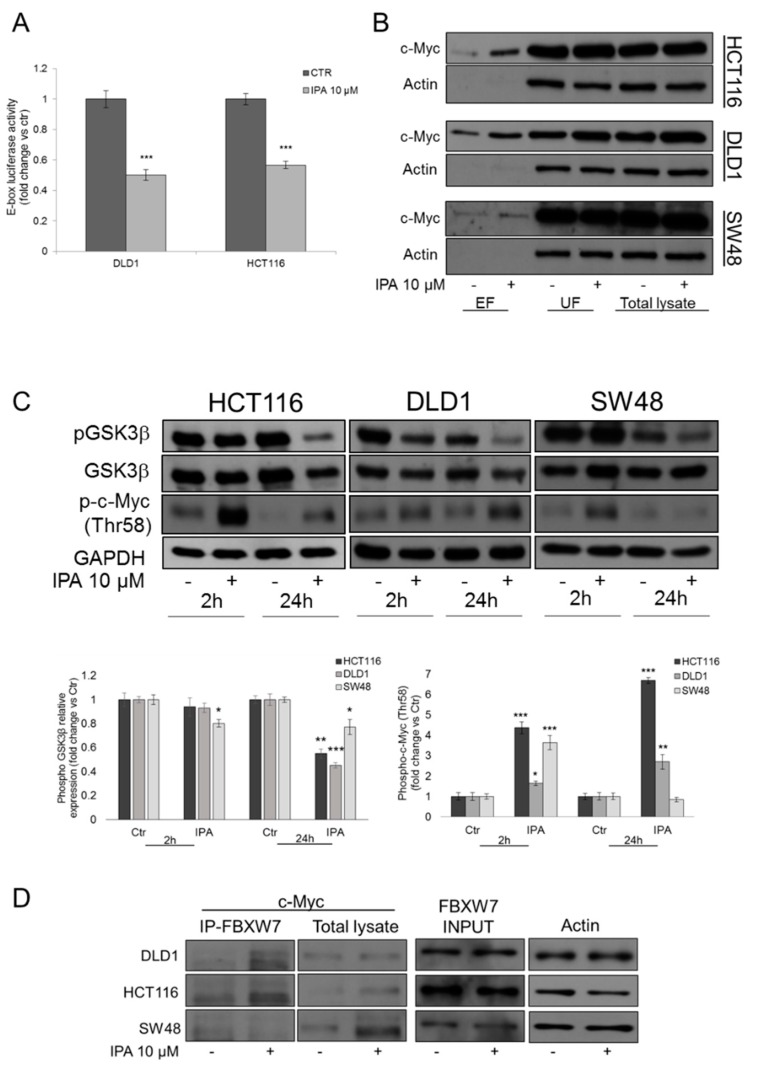
IPA affects c-Myc transcriptional activity through its ubiquitination. (**A**) Analysis of luciferase activity controlled by E-box elements in HCT116 and DLD1 cell lines. The histograms represent luciferase activity measured at 18 h from transfection of HCT116 and DLD1 cells transfected with reporter construct containing the E-box elements for c-Myc and treated with IPA 10 μM or vehicle. Firefly luciferase was normalized to *Renilla* luciferase reading and the data were plotted as fold change (mean ± SD of four independent experiments in triplicate; unpaired two tailed Student’s *t*-test *** *p* < 0.005) compared to control cells. (**B**) Representative western blot analysis of c-Myc expression in eluted fraction (EF) containing ubiquitinated proteins, unbound fraction (UF) depleted of ubiquitinated protein and whole protein lysates from HCT116, DLD1 and SW48 cells, treated with IPA 10 μM for 2 h. Actin was used as loading control. (**C**) Representative western blot and densitometry analysis of phosphorylated (Thr-58) c-Myc, phosphorylated (Ser9) GSK3β and total GSK3β in HCT116, DLD1 and SW48 cells treated with IPA 10 μM at the indicated time points. The histogram represents densitometry analysis of phosphorylated (Thr-58) c-Myc (right) or phosphorylated GSK3β expressed as fold change vs. total GSK3β (left), normalized vs. GAPDH. Data are expressed as mean ± SD of five independent experiments. * *p* < 0.05, ** *p* < 0.01, *** *p* < 0.005 vs. control. (**D**) DLD1, HCT116 and SW48 cells were treated with IPA or vehicle for 2 h. FBXW7 was Immunoprecipitated using anti-FBXW7 antibody. c-Myc expression was analyzed in FBXW7-IP fractions (to assess the reciprocal binding) and total cell lysates. FBXW7 protein levels before the IP was used as INPUT and Actin was used as loading control. Blots are representative of at least three independent experiments.

To exclude that the observed c-Myc increase was accompanied by a gain of its oncogenic activity, through luciferase assay we analyzed the effect of IPA on c-Myc promoter activation. In both HCT116 and DLD1 cells, transiently transfected with a reporter containing Transcriptional Responsive elements (TRE) for E-box binding element, after 24 h of treatment with IPA, luciferase activity was significantly inhibited of about 50%, compared to untreated cells (Figure 3A). This result demonstrated that the increase of c-Myc protein levels—observed after treatment with IPA—did not coincide with an increase of c-Myc/Max dimer transcriptional activity on E-box elements, suggesting that the elevated protein expression, could be due to additional mechanisms such as Post Transcriptional Modifications (PTMs) and in particular, given the increase of FBXW7 expression, to its ubiquitination.

### 2.4. IPA Induces FBXW7-Dependent c-Myc Ubiquitination

c-Myc ubiquitination occurs after GSK3β-mediated phosphorylation on Thr-58 residue, in *MYC* Box I, that allows FBXW7 to physically recognize c-Myc for subsequent ubiquitination [23]. We then analyzed the amount of phosphorylated GSK3β on inhibitory residue Ser-9. Accordingly to c-Myc transcriptional inactivation, IPA strongly reduced Ser-9 phosphorylation after 24 h of treatment in our three CRC models, but in particular in HCT116 and DLD1 cells (Figure 3C). In line with this, c-Myc phosphorylation (Thr-58) significantly increased after 2 h of treatment with IPA in all cell lines analyzed, but the induction persisted after 24h of treatment, in HCT116 and DLD1 cells only (Figure 3C).

To verify the hypothesis of IPA-mediated c-Myc ubiquitination, we pulled-down mono- and poly-ubiquitinated proteins, using a high-binding affinity matrix. As shown in Figure 3B, the amount of c-Myc was significantly raised in eluted fraction (EF) containing ubiquitinated protein, from IPA-treated DLD1 and even more in HCT116 cells, compared to EF from untreated cells; this turning out that, in *FBXW7* wild type cells, the increase of c-Myc protein expression was ascribable to its ubiquitination. In SW48, FBXW7^+/−^ cell line, the increase of ubiquitinated c-Myc was very slight and not significant, compared to wild type cells (Figure 3B and Supplementary Figure S2A).

Subsequently, we immunoprecipitated FBXW7 to assess its direct binding to c-Myc in CRC cell lines treated with the compound. Consistent with previous results, IPA strongly induced the binding of c-Myc and FBXW7 in both HCT116 and DLD1, but not in SW48 *FBXW7*^+/−^ cells, thus confirming that in wild type cells, the IPA-mediated ubiquitination was FBXW7-dependent (Figure 3D).

### 2.5. IPA Affects SREBP/FDPS Axis in FBXW7-Dependent Manner

**Figure 4:**
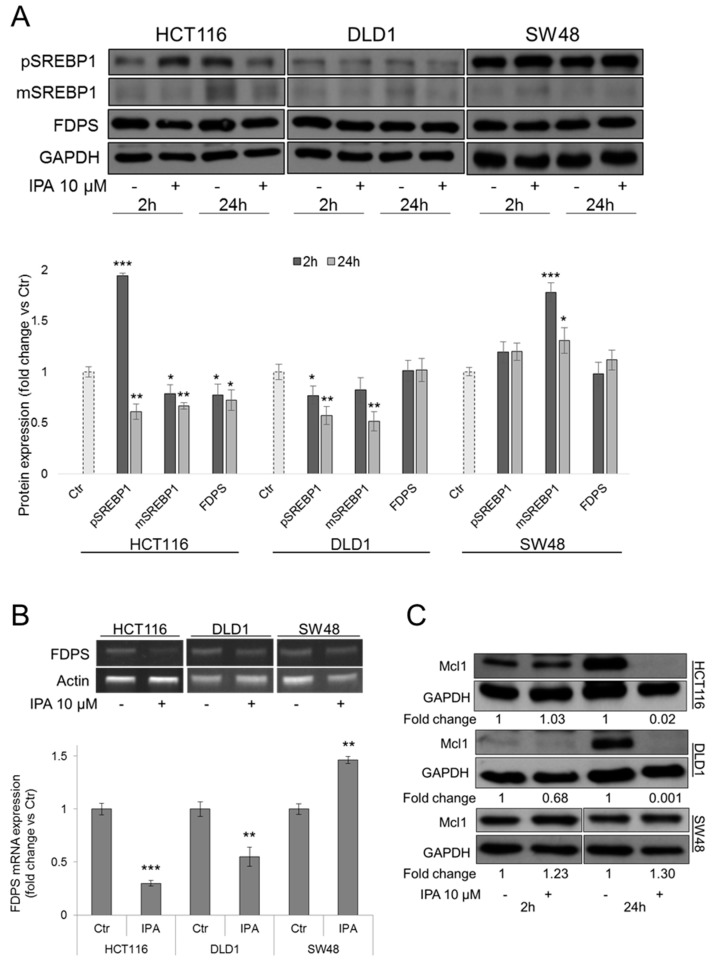
Modulation of SREBP/FDPS axis and Mcl1 substrate. (**A**) Representative western blot and densitometry analysis of SREBP1 precursor (pSREBP1), mature form (mSREBP1) and FDPS expression in HCT116, DLD1 and SW48 cells. GAPDH was used as loading control. Data are expressed as mean ± SD of six independent experiments. * *p* < 0.05, ** *p* < 0.01, *** *p* < 0.005 vs. control. (**B**) mRNA expression of *FDPS* gene determined by RT-PCR, in HCT116, DLD1 and SW48 cells after 24h of treatment with IPA 10 µM. Actin was used as loading control. The histograms report the quantification of the intensity bands expressed as mean ± SD of five independent experiments ** *p* < 0.01, *** *p* < 0.005 vs. control. (**C**) Representative western blot and densitometry analysis (fold change vs Ctr) of Mcl1 expression in HCT116, DLD1 and SW48 cells treated with IPA. GAPDH was used as loading control.

Sterol regulatory element-binding proteins (SREBPs) are transcription factors regulating the expression of genes involved in cholesterol and lipid biosynthesis, including FDPS gene. In CRC, downregulation of SREBPs arrests tumor growth, altering cellular metabolism. SREBPs become transcriptional active after cleavage in mature forms that, upon DNA binding, are degraded via proteasome. In this step, GSK3β catalyzes the phosphorylation of mature SREBP1, mediating the interaction with FBXW7 and thus its ubiquitination [24,25]. To evaluate the hypothesis of IPA-mediated regulation of FBXW7/SREBP/FDPS axis, we analyzed protein levels of both SREBP1 precursor and mature form. In HCT116 and DLD1 cells, IPA significantly decreased protein expression of both precursor and active mature form of SREBP1. In SW48 FBXW7^+/−^ cells, IPA failed to reduce SREBP1 expression, leading to its accumulation, as expected (Figure 4A). In our previous study, IPA was found to inhibit both the activity and mRNA expression of FDPS, in DLD1 cells [3]. Here we confirmed that, coherently with FBXW7-mediated SREBP1 downregulation, FDPS mRNA expression clearly decreased after treatment with IPA, in HCT116 and DLD1 cells. In SW48 cells, the failed degradation and then the accumulation of SREBP1 translated into a slight increase of *FDPS* gene expression (Figure 4A,B). To confirm that the inhibition of FDPS activity mediated by IPA in DLD1 is a common mechanism in CRCs, we analyzed—in HCT116 and SW48 cells—the levels of HDJ2, a chaperone protein that undergoes farnesylation-dependent processing. Unfarnesylated HDJ2, compared to farnesylated protein, runs with a higher molecular weight on SDS-PAGE gel, producing a mobility-shift identifiable by western blot analysis [26]. The mobility shift of unfarnesylated HDJ2 observed in both *FBXW7* wild type and mutant cells treated with IPA, suggested the early inhibition of FDPS activity, in agreement with results obtained previously in DLD1 cells [3] (Supplementary Figure S2B).

### 2.6. Synergistic Interaction of IPA and 5-FU

The involvement of FBXW7 in chemoresistance mechanisms is largely accepted. Recent study on human organoids showed that fbxw7^ΔG^ intestinal organoids are less sensitive to 5-fluorouracil (5-FU) treatment [27]. One of the hypothesis explaining resistance to oxaliplatin, 5-FU and tyrosine-kinase inhibitors (TKIs) in CRC and other tumors, involves the failure of Mcl1 degradation, the anti-apoptotic protein belonging to the BCL2 family, that is another well-known substrate of FBXW7 [16,21].

In *FBXW7* wild type cells, HCT116 and DLD1, but not in SW48 cells, IPA strongly reduced protein expression of Mcl1 (Figure 4C), suggesting an IPA-mediated control of chemoresistance. To this aim, using dedicated software CalcuSyn to calculate Combination Index (CI) and Dose Reduction Index (DRI), we analyzed the pharmacological interaction of IPA and 5-FU combination. As summarized in Table 1 and Supplementary Figure S3, the strongest synergistic interaction (CI 0.11–0.16) has been obtained in HCT116 cells by combined treatment with lowest concentrations of drugs, producing positive DRI. 

Unexpectedly, despite inhibition of Mcl1, in DLD1 cells IPA and 5-FU combination produced a moderate synergism or nearly additive effect, only for two combination with intermediate doses, thus antagonizing the effect of 5-FU or IPA used as single agents. Surprisingly, combined treatment of SW48 cells produced a synergistic interaction, with CI slight lower than HCT116, but higher than DLD1 (Table 1). CalcuSyn plots of dose vs. fraction Affected (FA) and FA-CI plots are showed in Supplementary Figure S3.

### 2.7. Identification of IPA Molecular Interactors

Chemical proteomic approach was used to identify potential molecular interactors of IPA in CRC cell lines. To this aim, DLD1 cells were exposed to the vehicle alone or to biotinylated IPA. Total protein extract obtained from CRC cells was fractionated and cytosolic and nuclear fractions were used for following experiments. As result—by means of binding studies to IPA-biotinylated, purification of the interactors and subsequent sequence analysis of databases—some proteins potentially able to bind biotinylated IPA were identified and summarized in Supplementary Table S1.

Curiously, among the most abundant interactors identified in the cytosolic fraction, α-Enolase (ENOA) oncoprotein plays a fundamental role in FBXW7-induced growth inhibition of colon cancer cells, being its direct substrate [28]. Nucleophosmin (NPM), identified in the nuclear fraction, is an oncoprotein mainly localized in nucleoli where it regulates genomic stability and DNA repair, histones assembly, centrosome duplication and cell cycle regulation. Like ENOA, also NPM physically binds FBXW7γ isoform, regulating its nucleolar localization [29]. The interactors Heterogeneus Nuclear Ribonucleoprotein R (HNRPR) and THO complex subunit 4, also found in the nuclear fraction, are both involved in mRNA processing and transport. Interestingly, histone H2B was the only protein detected in both cytosolic and nuclear extracts. Some of the other proteins identified, including NPM and histone H1.2, are direct partners of H2B.

### 2.8. IPA Increases Histone H2B Mono-Ubiquitination and RNF20 Protein Levels

**Figure 5:**
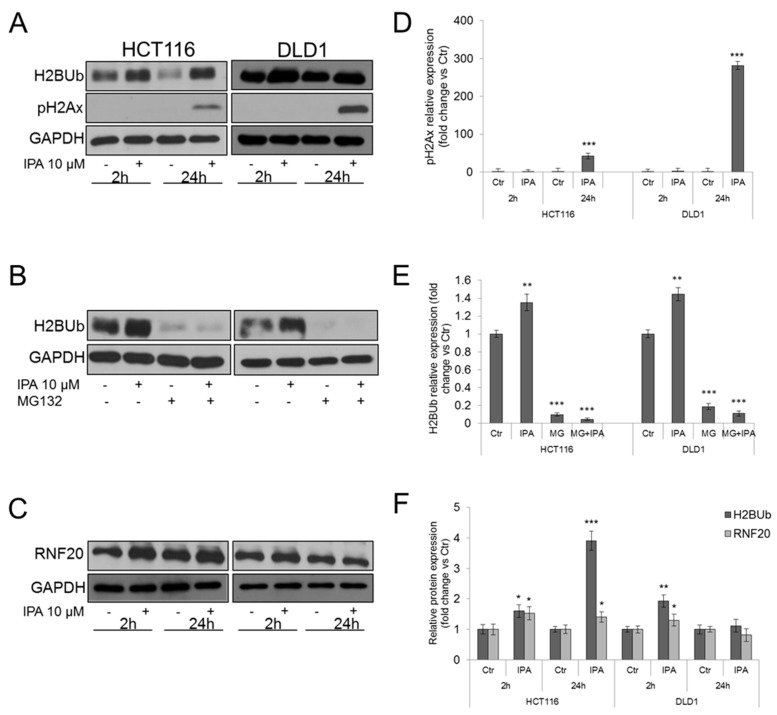
IPA regulates RNF20-mediated histone H2B ubiquitination. Western blot and densitometry analysis of (**A**,**D**) phosphorylated histone H2Ax and (**A**,**F**) Ubiquitinated histone H2B amount, in IPA-treated HCT116 and DLD1 cells; (**B**,**E**) histone H2B monoubiquitination after 2 h of treatment of HCT116 and DLD1 cells with IPA 10 µM alone or after 1 h of pre-treatment with MG132; (**C**,**F**) RNF20 in IPA-treated HCT116 and DLD1 cells. GAPDH was used as loading control. Data are expressed as mean ± SD of five independent experiments. * *p* < 0.05, ** *p* < 0.01, *** *p* < 0.005 vs. control.

Histone H2B undergoes mono-ubiquitination of Lys120 residue. H2BUb is a PTMs involved in several cellular functions, including carcinogenesis, and is catalyzed by RNF20/RNF40 E3 ubiquitin ligases complex [30]. Aimed to enforce our hypothesis and to examine the effect of IPA on other E3 ligases, we analyzed the amount of mono-ubiquitinated histone H2B through western blot assay of whole lysates from CRC cell lines. After treatment with IPA, H2BUb resulted significantly increased in both HCT116 and DLD1 cells (Figure 5A,F). H2BUb plays a well-known biological role in DNA damage response, since its co-localization with phosphorylated-ATM (ataxia telangiectasia mutated) and phosphorilated histone H2Ax (pH2Ax) on Double Strand Breaks (DSBs) sites is required for the recruitment of DNA damage repair factors, such as RAD51 and BRCA1 [31,32]. We then analyzed if the increased H2BUb correlated with pH2Ax and we found that, accordingly with apoptosis induction, pH2Ax significantly raised after 24 h of treatment (Figure 5A,D). However, the undetectable levels of pH2Ax observed after 2 h of treatment, suggested that the early increase of H2BUb could be associated to additional mechanisms.

Previous studies reported that proteasome inhibition strongly and rapidly reduces ubiquitinated histones amount. In particular, proteasome inhibition causes the accumulation of poly-ubiquitin chains, inhibiting their recycling. Therefore, depletion of cytoplasmic and nuclear free ubiquitin pools occurs [33]. Hence, in our models, treatment with the proteasome inhibitor MG132 strongly reduced the amount of H2BUb (Figure 5B,E). Interestingly, pre-treatment with MG132 made IPA unable to induce H2BUb mono-ubiquitination (Figure 5B,E), suggesting that it could act as direct modulator of ubiquitin conjugation on target proteins, corroborating our previous findings. Supporting this hypothesis, we analyzed protein expression levels of RNF20. In HCT116 cells (*RNF20* wild type) IPA significantly induced RNF20 protein expression after 2 h and 24 h of treatment, while in DLD1 cells (*RNF20* mutant) a slight but significant increase of RNF20 protein expression was detected after 2 h of treatment, but not sustained (Figure 5C,F). The increase of RNF20 expression mirrors the trend of H2BUb, after treatment with IPA.

### 2.9. IPA Induces Regression of CRC In Vivo

**Figure 6:**
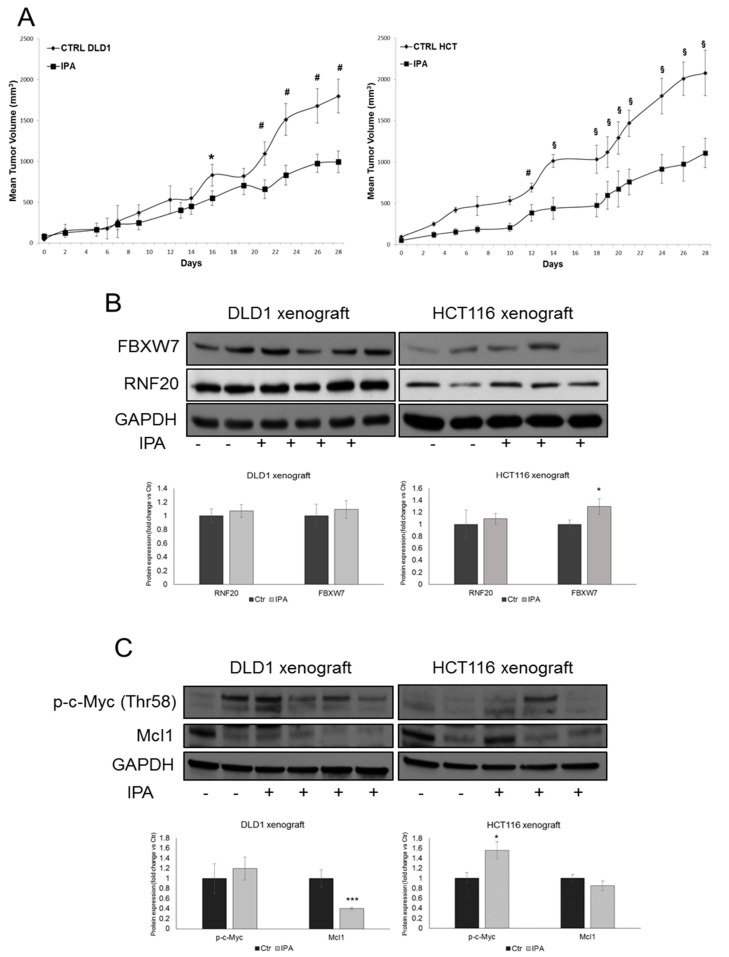
IPA reduces CRC growth in vivo. (**A**) Tumor volume growth curve of DLD1-xenograft (left panel, *n* = 20 mice, 10 mice in each group) and HCT116-xenograft (right panel; *n* = 20 mice, 10 mice in each group) after peri-tumoral injection of IPA. Growth retardation by the compound was statistically significant for all time points labelled with * (one-way ANOVA *p* < 0.05), with # (one-way ANOVA *p* < 0.01) and § (one-way ANOVA *p* < 0.001). Western blot and densitometry analysis of FBXW7, RNF20 (**B**), phosphorylated-c-Myc (Thr58) and Mcl1 (**C**) in whole lysate from representative resected tumor tissues. GAPDH was used as loading control. Data are expressed as mean ± SD of at least four independent experiments. * *p* < 0.05, *** *p* < 0.005 vs. control group.

We tested IPA efficacy in vivo in subcutaneous HCT116 and DLD1 xenograft models. Tumor cell suspensions were injected s.c. into 20 female SCID mice, respectively, and when the tumor reached approximately the size of 50–70 mm^3^, 10 mice in the treated group received the peri-tumoral injection of IPA, while 10 mice in the control group received vehicle alone, three times a week for 4 weeks. The tumor sizes have been recorded on the first day of IPA treatment (day 0) and bi- or three-weekly at the indicated time points. Mice in IPA groups developed much smaller tumors (Figure 6A). In particular, starting from the 16th and the 12th day of treatment of DLD1 and HCT116 xenografts, respectively, ANOVA analysis indicated a significant smaller tumor size in treated groups compared with animals in the control groups (* *p* < 0.05; # *p* < 0.01; § *p* < 0.001) (Figure 6A).

Analysis of total protein extracts from tissue specimens evidenced that—despite the high tumor samples heterogeneity—the expression of FBXW7 was significantly induced in the IPA-treated HCT116 xenografts, compared to control tumors. Although did not reach statistical significance, similar results have been obtained in DLD1 xenografts (Figure 6B). Corroborating the in vivo regulation of FBXW7, Mcl1 anti-apoptotic protein decreased in both DLD1 and HCT116 xenografts treated with IPA. Moreover, c-Myc phosphorylation on Thr-58 residue increased in IPA-treated xenografts compared to control group, with a higher and significant effect in HCT116 xenograft (Figure 6C). Unfortunately, although the slight increase, the expression of RNF20 was not significantly modulated (Figure 6B).

## 3. Discussion

In vitro and in vivo antitumor effect of IPA have been commonly, but not exclusively, associated with FDPS inhibition, a key enzyme involved in mevalonate pathway—responsible for cholesterol biosynthesis and protein prenylation—widely involved in cancer [1,5,34]. However, the exact molecular mechanism underlying its pleiotropic effects remains a question mark.

FBXW7 is the F-box protein that works as adaptor of SCF E3 ligase [35]. Inactivating mutations of FBXW7 have been found in a large spectrum of cancer types, including CRC, and its role as tumor suppressor is widely reported [14,15,17].

The finding that IPA modulates E3 ubiquitin ligases, provides a believable link between the dissimilar effects observed in different cancers. The identification of FBXW7 as molecular target of IPA, enforces our previous reports and suggests that FDPS inhibition could be a direct consequence of IPA-mediated regulation of the FBXW7/SREBP1/FDPS axis, in a FBXW7-dependent manner. Several studies highlighted the interplay between mevalonate pathway and c-Myc regulation. Statins —the well-known inhibitors of endogenous cholesterol biosynthesis [36]—reduce the growth of Brain Tumor Initiating Cells (BTICs), where mevalonate pathway genes, including *FDPS*, are strongly upregulated, through a feed-forward loop between c-Myc and the mevalonate pathway [37]. Furthermore, some reports suggested that cholesterol—one of the end products of mevalonate pathway—or its metabolite 27-hydroxycholesterol (27-HC) control cancer cell proliferation through downregulation of FBXW7. In particular, in breast cancer cells, 27-HC suppresses *FBXW7* transcription leading to an increase of c-Myc protein stability [38,39].

Here we found that IPA is able to transcriptionally inactivate c-Myc and to upregulate FBXW7-c-Myc binding in *FBXW7* wild type cells but not, as expected, in SW48 mutant cells where—despite IPA mediated antiproliferative and pro-apoptotic effects (Supplementary Figure S1)—c-Myc accumulation occurs in a time-dependent manner. Given its central role in cell metabolism and proliferation, c-Myc abundance is strictly regulated at multiple steps. Therefore, especially in cancer cells, c-Myc undergoes a rapid turnover ensuring its availability and adequate protein levels. This consideration could explain why—although c-Myc-Thr58 phosphorylation, subsequent ubiquitination and transcriptional inhibition clearly suggested the IPA-mediated regulation of c-Myc turnover—we did not observe the complete abrogation of its protein levels in our CRC models. Further, the different trend of modulation observed in DLD1 and HCT116 cells is not surprising, since the half-life of c-Myc could be a result of genotypes. For instance, in CRC, Ubiquitin Specific Peptidase 9, X-Linked (USP9x) deubiquitinase positively regulates FBXW7 stability, antagonizing its ubiquitylation and proteasomal degradation. While HCT116 cell line carries wild type *USP9x* gene, DLD1 cells displays a mutation in its catalytic domain [40]. In addition, as previously debated, DLD1 cells harbor a high percentage of *FBXW7* promoter methylation [22].

On the other hand, the upregulation of FBXW7 observed in DLD1 and the inactivation of Myc responsive elements, suggests transcriptional and/or epigenetic IPA-mediated mechanisms. Moreover, both H2BUb and FBXW7 are involved in the regulation of various mediator complexes that physically link transcription factors to transcriptional machinery and RNA Polymerase II [41,42].

So far, some studies provided different evidence about the molecular mechanism of IPA and many of these substantially agree with data obtained from the present work. We previously reported that, in melanoma cells, IPA exerts a dual role on autophagy regulation [7,8]. Cullin-RING ligases regulate autophagy acting at multiple levels of transduction pathway. The crucial regulators of autophagy, such as the Ras inhibitor NF1 (Neurofibromin 1) and mTOR, are substrates of SCF^FBXW7^ [43]. Furthermore, a recent work examined the role of H2BUb in the epigenetic regulation of autophagy. Alterations of H2BUb levels correlate with global changes in autophagy related genes and the H2BUb levels are inversely correlated to the induction of autophagy [44]. These findings strongly support results here showed, since we observed an early increase of H2BUb and RNF20 E3 ligase levels, in IPA treated cells, followed by induction of apoptosis. Even though underling mechanisms triggering H2BUb and its biological relevance have not been extensively evaluated in this study, there is a positive match with the other results. As debated, FBXW7 regulates several transcription factors and epigenetic enzymes taking part in biological processes, such as DNA damage, in which H2BUb is firmly involved. Thus, the increase of H2BUb should not be considered an additional mechanism, but probably a related consequence of FBXW7 modulation. Besides, RNF20 and SCF^FBXW7^ belong to RING-domain ligases family and it is conceivable that more than one ligase in a subfamily can be targeted by the compound.

Recently, we also reported the first evidence on IPA ability to induce ubiquitination. In glioma in vitro models, IPA induces EGFR ubiquitination through the upregulation of c-Cbl, an E3 ligase belonging to RING—domain ligase family such as SCF^FBXW7^ and RNF20 [10]. Several evidence suggested the E3 ligases, including SCF^FBXW7^, as target to overcome temozolomide resistance in GBM [45] and TKIs resistance in different cancers. In both non–small cell lung cancer (NSCLC) and CRC, Mcl1 accumulation—frequently ascribable to FBXW7-inactivating mutations—leads to intrinsic and acquired resistance to oxaliplatin, 5-FU and several TKIs [16,21,27,46]. In GBM, *FDPS* silencing brings about a reduction of Mcl1 levels [47]. This result, together with data showed here, corroborates the potential interplay between mevalonate pathway, Mcl1 and E3 ubiquitin ligases, in particular SCF^FBXW7^. A limit of our study is the lack of FBXW7 specific inhibition experiments. However, the absence of selective pharmacological inhibitors and the expression of different FBXW7 isoforms, make such tests difficult to perform and do not necessarily could provide unequivocal and exhaustive results. On the other hand, the evidence that so far IPA was reported as FDPS inhibitor and the data on FBXW7/SREBP/FDPS axis, showed here, fit reciprocally supporting our conclusions.

Combined treatments reveal an unexpected but plausible evidence. In HCT116 cells, the combined use of IPA and 5-FU produced the strongest synergistic interaction, with a very low CI and a DRI corresponding to the lowest concentrations of the drugs. In SW48 cells, albeit the *FBXW7* mutation affects Mcl1 degradation, the combination of IPA and 5-FU was synergistic starting from intermediate concentrations. Unexpected, in DLD1 cells combination of IPA and 5-FU predominantly antagonized, at least for the time point and doses analyzed. Bracht and colleagues [48], classified 5-FU sensitivity in a panel of CRC cell lines, based on mismatch repair (MMR) status. Although our three in vitro models share MMR deficiency, they found that SW48 cells show an intermediate 5-FU sensitivity, compared to the resistant phenotype of HCT116 and even more of DLD1 cells, that displays the highest GI_50_. This concurs with our results and *FBXW7* wild type in HCT116 cells could dictate the synergism with 5-FU. Beyond the greater basal sensitivity to 5-FU of SW48 cells, the better effect of the combination in these cells could be ascribable to *TP53* gene status. In particular, while DLD1 cells harbor mutant p53, both HCT116 and SW48 display wild type *TP53* [49]. p53 limits the effects of FBXW7 loss and its mutation is frequently associated with hypermethylation of FBXW7 promoter regions, as occurs in DLD1 cell line. Loss of both p53 and FBXW7 causes chromosomal instability (CIN) of intestinal cancer, accelerating tumorigenesis and conferring advanced phenotypes [50,51]. This probably explains the antagonistic interaction of IPA and 5-FU in DLD1 cells, compared to wild type *TP53* cell lines. As well, the response to 5-FU and generally to chemotherapics or their combination can be influenced by a multitude of genetic variables. On the other hand, this is what occurs in patients, where the high intra- and inter- tumor heterogeneity, dictates the high variability of the patient’s response.

The ability to increase sensitivity to 5-FU is an intriguing property of IPA. Recently, in HCT-8R—a *TP53* wild type CRC cell line insensitive to 5-FU—the suppression of the checkpoint kinase 1 (CHK1) pathway, induced by WNT/β-catenin activation, has been described as a potential mechanism associated to the acquired resistance to chemotherapy drugs. The 5-FU-resistant phenotype correlated with an imbalance of histone acetylation/deacetylation, mainly in H3K14 and H3K27, induced by WNT/β-catenin activation [52]. These pieces of evidence substantially strengthen our results taking into account that in CRC harboring stabilizing mutation of β-catenin (such as in HCT116 and SW48) and the genetic profile of metastatic CRC, poorly responsive to chemotherapies [53] the ability of IPA to synergize with 5-FU could represents a valuable effect and potentially a good therapeutic option. Our results suggest that IPA acts at multiple steps of oncogenic pathways transduction directly affecting ubiquitin ligases and, probably as a consequence, regulating epigenetic and/or transcriptional pathways. This hypothesis, although today still speculative, springs from evidence obtained in melanoma cells, where IPA is rapidly converted in N6-isopentenyladenosine monophosphate (iPAMP) active form [7]. Thus, it is likely that, as an AMP mimetic, IPA could act upstream of catalytic triad, on E1 or E2 ligases, where ATP and AMP balance rules the adenylation required for ubiquitin activation. Moreover, proteomic studies showed here suggested a physical interaction between IPA and some E3 ligases substrates (Supplementary Table S1). However, since antitumor and pro-apoptotic effect was verified also in FBXW7^+/-^ SW48 cells, it is strongly advisable to dissect the specificity of IPA and to clarify if its efficacy is mediated by activities restricted to selected ligases or selected subfamilies such as RING-domain ligases family, to which RNF20, CBL and SCF^FBXW7^ belong.

## 4. Material and Methods

### 4.1. Cell Cultures, Reagents and Proliferation Assay

Human CRC cells DLD1 (ICLC Cat# HTL95011, RRID:CVCL_0248), HCT116 (ICLC Cat# HTL95025, RRID:CVCL_0291) and SW48 (ICLC Cat# HTL99020, RRID:CVCL_1724) were obtained from the ICLC (IST, Genoa, Italy) and routinely grown in monolayers in RPMI-1640, McCoy’s and DMEM-F12 medium, respectively, supplemented with 10% heat inactivated FBS, 2 mM L-glutamine, 50 ng/mL streptomycin, 50 units/mL penicillin. Cell lines were maintained at 37 °C in a humidified 5% CO_2_ atmosphere and cultured for no more than four passages and no longer than three weeks, for each experiment performed. Cell lines are biannually tested for mycoplasma. All reagents for cell culture, N6-isopentenyladenosine (IPA), 5-fluorouracil (5-FU) and MG132 inhibitor (dissolved in DMSO) were purchased from Sigma–Aldrich (St. Louis, MO, USA). Cell proliferation was evaluated through BrdU incorporation measurement, through a colorimetric ELISA kit (Roche Diagnostics GmbH, Mannheim, Germany), as previously described [54]. All experiments were performed in triplicate, and the relative cell proliferation was expressed as a fold change *versus* untreated control cells.

### 4.2. Cytofluorimetric Analysis

To assess the effect of IPA on cell cycle profile, HCT116 and DLD1 cells were plated and treated with vehicle or IPA 10 µM for 24 h. At the end of treatment, cells were collected, fixed in 70% ethanol and kept at −20 °C overnight. Propidium iodide (PI; 50 µg/mL) was added to the cells for 15 min and for each sample at least 10,000 events were acquired. The analysis was performed using ModFit LT v3.2 software (Verity Software House, Inc., Topsham, ME, USA, RRID:SCR_016106).

Annexin V/PI double staining was used to examine apoptosis induction. DLD1 cells treated with vehicle or IPA 10 µM for 24 h, were harvested through trypsinization and stained with FITC-conjugated Annexin V for 20 min at room temperature and then with PI for additional 15 min in the dark. At least 10,000 events were acquired and the analysis was performed with FlowJo v10.5 software (Becton, Dickinson and Company, Franklin Lakes, NJ, USA, RRID:SCR_008520).

### 4.3. Western Blot Analysis

Protein expression from whole cell lysates was analyzed as previously described [55]. Briefly, CRC cells, treated with IPA 10 µM or vehicle alone, were lysed in RIPA buffer (50 mM Tris–HCl pH 8.0 buffer containing 150 mM NaCl, 1% Nonidet P-40, 2 mg/mL aprotinin, 1 mg/mL pepstatin, 2 mg/mL leupeptin, 1 mM Na_3_VO_4_). Total protein extracts from tissue specimens were obtained by disrupting frozen tumor pieces by gentle homogenization (Potter-Elvehjem Pestle) in cold RIPA buffer. Protein concentration was determined through Bradford method. Cell or tissue lysates were subjected to 10–12% SDS-PAGE. Gels were electroblotted into nitrocellulose membranes that were probed with the following primary antibodies: anti-Caspase 3 polyclonal (1:1000; Cat# 9662, RRID:AB_331439); rabbit anti-Cleaved Caspase-3 monoclonal (1:1000; Cat# 9664, RRID:AB_2070042); rabbit anti-Cleaved PARP monoclonal (1:1000; Cat# 5625, RRID:AB_10699459); rabbit anti-GAPDH monoclonal (1:5000; Cat# 2118, RRID:AB_561053); rabbit anti-Phospho-GSK-3beta monoclonal (1:1000; Cat# 9323, RRID:AB_2115201); rabbit anti-GSK-3beta monoclonal (1:1000; Cat# 9315, RRID:AB_490890); rabbit anti-Mcl-1 monoclonal (1:1000; Cat# 5453, RRID:AB_10694494); rabbit anti-Ubiquityl-Histone H2B monoclonal (1:1000; Cat# 5546, RRID:AB_10693452); rabbit anti-Phospho-Histone H2A.X polyclonal (1:1000; Cat# 2577, RRID:AB_2118010) purchased from Cell Signalling Technology (Beverly, MA, USA). Mouse anti-c-Myc monoclonal (1:500; Cat# sc-40, RRID:AB_627268); mouse anti-SREBP-1 monoclonal (1:500: Cat# sc-13551, RRID:AB_628282) purchased from Santa Cruz Biotechnology (Santa Cruz, CA, USA). Rabbit Anti-Fbxw7 polyclonal (1:1000; Cat# ab109617, RRID:AB_2687519); rabbit anti-Actin polyclonal (1:10000; Cat# ab37063, RRID:AB_722537); mouse anti-c-Myc monoclonal (1:1000; Cat# ab32, RRID:AB_303599); rabbit anti FDPS polyclonal (1:1000; Cat# ab153805); rabbit anti-RNF20 polyclonal (1:1000; Cat# ab32629, RRID:AB_873630); rabbit anti-HDJ2 monoclonal (1:1000; Cat# ab126774, RRID:AB_11127505) purchased from Abcam (Cambridge, UK). Rabbit anti-Phospho-c-Myc polyclonal (1:1000; Cat# PA5-36673, RRID: AB_2553655) was purchased from Thermo Fisher Scientific (Waltham, MA, USA). After incubation with primary antibody, membranes were probed with peroxidase-coniugated secondary antibodies. The goat anti-rabbit secondary antibody (1:5000; Cat# ab6721, RRID: AB_955447) and goat anti-mouse secondary antibody (1:5000; Cat# ab6789, RRID: AB_955439) were purchased from Abcam. To detect proteins, membranes were incubated with an enhanced chemiluminescence reagent (GE Healthcare, Hilden, Germany) and exposed to X-ray film (Santa Cruz Biotechnology, Dallas, TX, USA). Immunoreactive bands were quantified with ImageLab v4.0 analysis software (Bio-Rad, Hercules, CA, USA, RRID:SCR_014210). 

### 4.4. Luciferase Assay

Luciferase assay was performed with modified protocol previously described [53]. Briefly, DLD1 and HCT116 cells were transiently cotransfected with the Myc/Max firefly luciferase construct (100 ng) and the *Renilla* luciferase vector (10 ng) to normalize transfection efficiency (Cignal Myc Reporter assay kit, QIAGEN, Hilden, Germany). After 18 h from transfection, a dual luciferase assay was performed according to manufacturer’s instruction (Promega, Madison, WI, USA). Luciferase readings were measured using an EnSpire-2300 luminometer (Perkin Elmer, Waltham, MA, USA). Data were represented as relative luciferase activity, obtained by the ratio of firefly values (promoter reporter) to *Renilla* values (control reporter). Experiments were repeated at least three times, and values are expressed as the mean ± SD.

### 4.5. Isolation and Enrichment of Ubiquitinated c-Myc

Isolation of ubiquitinylated proteins was performed using UBIQAPTURE-Q kit (Enzo Life Science, Lausen, Switzerland) following manufacturer’s instructions. Briefly, total protein extracts were obtained using buffer A (20 mM TRIS, 100 mM NaCl, 5 mM EDTA) containing protease/phosphatase inhibitors. 25 µg of total lysate added to the affinity matrix and incubated at 4 °C overnight. Samples were centrifuged to collect the supernatant representing the ‘unbound fraction’ (UF). The elution of ubiquitin-protein conjugates (EF) was carried out by adding SDS-PAGE gel loading buffer 1× (250 mM TRIS, pH 6.8, 15% SDS, 50% glycerol, 25% β-mercaptoethanol, 0.01% bromophenol blue), followed by mixing at room temperature for 5 min, heating to 95 °C for 10 min and clarification. Equal volumes of the obtained ‘eluted fractions’, ‘unbound fractions’ and original lysates, prepared likewise, were subjected to western blotting analysis, using anti-c-Myc primary antibody. To assess the efficiency of ubiquitinated protein isolation, control lysate supplied in the kit was processed in parallel. The efficiency of ubiquitinated protein enrichment is represented in Supplementary Figure S4.

### 4.6. Immunoprecipitation Assay

The IP experiments were performed using the Pierce Classic IP Kit (Thermo Scientific, Rockford, IL, USA). About 700 µg of whole lysates obtained in IP Lysis/Wash Buffer (25 mM TRIS, 150 mM NaCl, 1mM EDTA, 1% NP-40, 5% glycerol, pH 7.4) containing protease/phosphatase inhibitors, were pre-cleared with the Control Agarose Resin for 1 h. The cleared lysates were combined with 7 µg of anti-FBXW7 primary antibody and incubated overnight at 4 °C with gentle mixing to form the immune complexes. Subsequently, 20 µL of the Pierce Protein A/G Agarose resin suspension were placed into the spin columns provided, washed twice with ice-cold IP Lysis/Wash Buffer and then incubated with the antibody/lysate samples for two hours. Columns were spun by centrifuge at 1000× g for 1 min to collect the flow-through and the resin was washed three times with IP Lysis/Wash Buffer and once with 1× Conditioning Buffer. Elution of immune complexes was carried out by adding 50 µL of 2× Non-reducing Lane Marker Sample Buffer with a final concentration of 20 mM DTT and incubated at 100 °C for 10 min. Eluates were collected by spinning and applied to SDS-PAGE gel.

### 4.7. RNA Extraction and Semi-Quantitative RT-PCR

Total RNA extraction, cDNA synthesis and reverse-transcription PCR were performed as previously described [56]. Primer pairs specific to human *FDPS* (5′-*CAGATCTGCTGGTATCAGAA*-3′ forward and 5′-*GTGCTCCTTCTCGCCATCAAT*-3′ reverse) or to human *Actin B* (5′-*ACTGGG ACGACATGGAGAA*-3′ forward and 5′-*ATCTTCATGAGGTAGTCAGTCA*-3′ reverse) were used. All reactions were performed at least in triplicate in three independent experiments; the PCR products were quantified with ImageLab v4.0 analysis software (Bio-Rad) and results were normalized to those obtained from *Actin B*.

### 4.8. Drug Combination Analysis and Cell Viability Assay

Drug combination analysis was performed as previously described [55]. Briefly, HCT116, DLD1 and SW48 cells were exposed to various concentrations of IPA and/or 5-FU, to evaluate cells viability, colorimetric MTT assay was used. Pharmacological interaction between IPA and 5-FU, were calculated using CalcuSyn v2.0 software (BioSoft, Ferguson, MO, USA), a dedicated software based on the Chou-Talalay method. This allows to calculation of two parameters: Combination Index (CI)—to define synergism (CI < 1), additive effect (CI = 1) and antagonism (CI > 1) – and Dose Reduction Index (DRI) [57]. Assessment of drug interactions was performed calculating CI after treatment for 48 h with IPA and 5-FU in combination (constant molar ratio 1:2.5) and as single drugs, ranging from 0.08 µM to 10 µM of IPA and from 0.2 µM to 25 µM of 5FU.

### 4.9. Chemical Proteomics

A chemical proteomic approach, able to define cellular targets of selected drug candidates based on compound-immobilized affinity chromatography, was used as previously described [58,59] to identify potential molecular interactions of IPA. To that aim, cytokinin was biotynilated through a two-step modification procedure; briefly, IPA was incubated with N(3Br-propyl)phthalimide in N,N-dimethylformamide in the presence of 1 mM NaH. The resulting product was than underwent hydrazinolysis, followed by incubation with biotin-aldehyde. The product was purified by HPLC-UV and its structure and purity were confirmed by mass spectrometry. DLD1 were incubated with 10 μM biotinylated IPA for two hours at 37 °C and then underwent protein extraction procedure. Subcellular fractionation of nuclear and cytosolic protein from treated and control cells was obtained using NE-PER^®^ Nuclear and Cytoplasmic Extraction Reagents (Thermo Scientific) as described previously [53]. Clarified whole cell lysates (100 μg) were incubated with IPA-loaded or with control resin (for 2 h at 25 °C) and interacting proteins were eluted and analyzed as reported elsewhere [58].

### 4.10. In Vivo Tumor Growth Assay

Forty female SCID mice (SHO, 6–8 weeks old; Cat# CRL:474, RRID: IMSR_CRL:474), obtained from Charles River Laboratories (Sulzfeld, Germany), were maintained and nourished as described elsewhere [53]. All the in vivo procedures were in accordance with the Institute for Laboratory Animal Research Guide for the Care and Use of Laboratory Animals. The experimental protocols received the approval from the Ethical Committee of the Italian Board of Health (prot. n. 0,031,993 and n. 0031994, 6 June 2013).

HCT116 cells (2 × 10^6^/150 μL/mouse) or DLD1 cells (2.5 × 10^6^/150 μL/mouse) were implanted subcutaneously into the right flank region of 20 mice, respectively, and allowed to grow to the size of approximately 50–70 mm^3^. Mice were then randomly assigned to vehicle control (CTRL; *n* = 10 mice for each xenograft) or treated (IPA; *n* = 10 mice for each xenograft) groups.

IPA (0.016 mg/dose) or vehicle alone were administered three time a week by peri-tumoral injection for 4 weeks. Mice were daily monitored for clinical signs and mortality. Tumor volumes were determined by two dimensional caliper measurements and calculated using a standard hemi ellipsoid formula: [length (mm) × width (mm)^2^]/2. At the end of the study, mice were humanely euthanized and tumors were resected for further analysis.

### 4.11. Statistical Analysis

Results are expressed as mean ± standard deviation (SD) of three independent experiments at least. Statistical differences between the treatment and the control groups were evaluated by one-way ANOVA for in vivo experiments. Student’s *t* test was performed for all in vitro experiments. A *p* value less than 0.05 was considered statistically significant.

## 5. Conclusions

The identification of molecules like IPA—able to upregulate, both in vitro and in vivo, the oncosuppressor FBXW7—is strongly advisable since its restoration can be achieved only through indirect approaches [60]. In CRC, FBXW7 represents an interesting therapeutic target in the light of its involvement in the regulation of several cancer-related pathways, including chemoresistance.

Overall, our results propose a potential new mechanism, related to evidence recently reported for IPA-mediated antitumor effects, but also able to unify some seeming discrepancies observed in different tumors. Furthermore, mevalonate pathway and FBXW7 crosstalk could represents an appealing novel target for therapeutic intervention. However, taking into account that the genotype seems to strongly influence the response to IPA and its combination with 5-FU, a wide correlation is needed to pinpoint the better therapeutic strategy for this compound, in different tumor types.

## Figures and Tables

**Table 1:** IPA synergizes with 5FU. Fraction Affected (FA), Combination Index (CI) and Dose Reduction Index (DRI) for IPA and 5FU combination (constant molar ratio 1:2.5) in HCT116, DLD1 and SW48 cells.

**HCT116**						
**Concentration**		**FA IPA + 5FU**	**CI and Symbols**		**DRI**	
**IPA (µM)**	**5FU (µM)**				**IPA**	**5FU**
0.08	0.2	0.25	0.11	++++	38.64	11.92
0.16	0.4	0.29	0.16	++++	21.68	8.48
0.31	0.78	0.29	0.31	+++	11.31	4.52
0.63	1.56	0.42	0.33	+++	7.39	5.2
1.25	3.13	0.48	0.5	+++	4.18	3.78
2.5	6.25	0.53	0.79	++	2.35	2.71
5	12.5	0.67	0.95	±	1.56	3.21
10	25	0.79	1.13	−	1.1	4.57
**DLD1**						
**Concentration**		**FA IPA + 5FU**	**CI and Symbols**		**DRI**	
**IPA (µM)**	**5FU (µM)**				**IPA**	**5FU**
0.08	0.2	0.19	116.42	−−−−−	23.89	0.01
0.16	0.4	0.35	1.69	−−−	17.98	0.61
0.31	0.78	0.4	0.99	±	10.3	1.12
0.63	1.56	0.46	0.7	++	5.66	1.91
1.25	3.13	0.45	1.54	−−−	2.8	0.85
2.5	6.25	0.48	1.78	−−−	1.49	0.9
5	12.5	0.59	1.23	−−	0.93	6.5
10	25	0.83	1.21	−−	0.82	3374.52
**SW48**						
**Concentration**		**FA IPA + 5FU**	**CI and symbols**		**DRI**	
**IPA (µM)**	**5FU (µM)**				**IPA**	**5FU**
0.08	0.2	0.21	0.43	+++	6.154	3.701
0.16	0.4	0.21	0.93	±	2.977	1.67
0.31	0.78	0.26	1.02	±	1.991	1.926
0.63	1.56	0.42	0.58	+++	2.091	9.793
1.25	3.13	0.6	0.49	+++	2.12	43.917
2.5	6.25	0.75	0.5	+++	2.03	164.813
5	12.5	0.86	0.48	+++	2.093	779.084
10	25	0.89	0.72	++	1.388	936.115

Symbols describe the pharmacological interaction for each combined dose, based on CI obtained from CalcuSyn v2.0 software (++++ Strong synergism; +++ Synergism; ++ Moderate synergism; ± Nearly additive; − Slight antagonism; −− Moderate antagonism; −−− Antagonism; −−−−− Very strong antagonism).

## Supplementary Materials

The following are available online at https://www.mdpi.com/2072-6694/11/10/1456/s1, Figure S1: IPA arrests cells proliferation and induces apoptosis in SW48 cells, Figure S2: HDJ2 mobility shift, Figure S3: Drug combination analysis, Figure S4: Efficiency of ubiquitinated protein enrichment, Figure S5: Whole western blots, Table S1: Molecular interactions of IPA.
