# Supplementary material for: N6-Isopentenyladenosine Inhibits Colorectal Cancer and Improves Sensitivity to 5-Fluorouracil Targeting FBXW7 Tumor Suppressor
Source: Cancers (Basel). 2019 Sep 28;11(10):1456. doi: 10.3390/cancers11101456 (PMC6826543; doi:10.3390/cancers11101456)
Supplement: Supplementary file 1 [file cancers-11-01456-s001.pdf]

Supplementary Materials

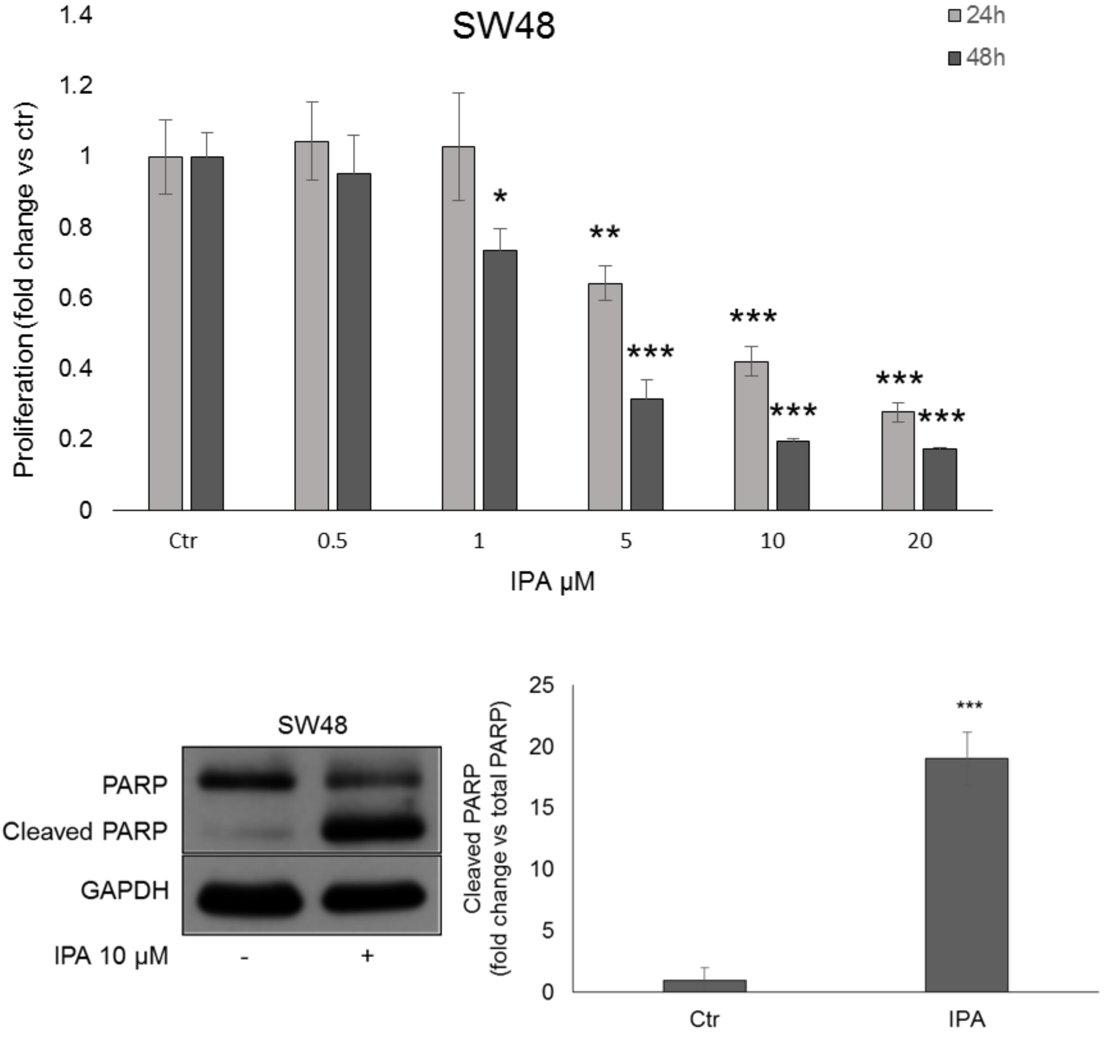

Figure S1. IPA arrests cells proliferation and induces apoptosis in SW48 cells. BrdU incorporation assay in SW48 cells treated with indicated concentrations of IPA, for 24 h or 48 h (upper panel). Representative western blot and densitometry analysis (lower panel) of Cleaved and total PARP in SW48 treated for 24 h with IPA 10 µM. Data are expressed as mean ± SD of 5 independent experiments in triplicate (* $p < 0.05$, ** $p < 0.01$, *** $p < 0.005$ vs. control). GAPDH was used as loading control.

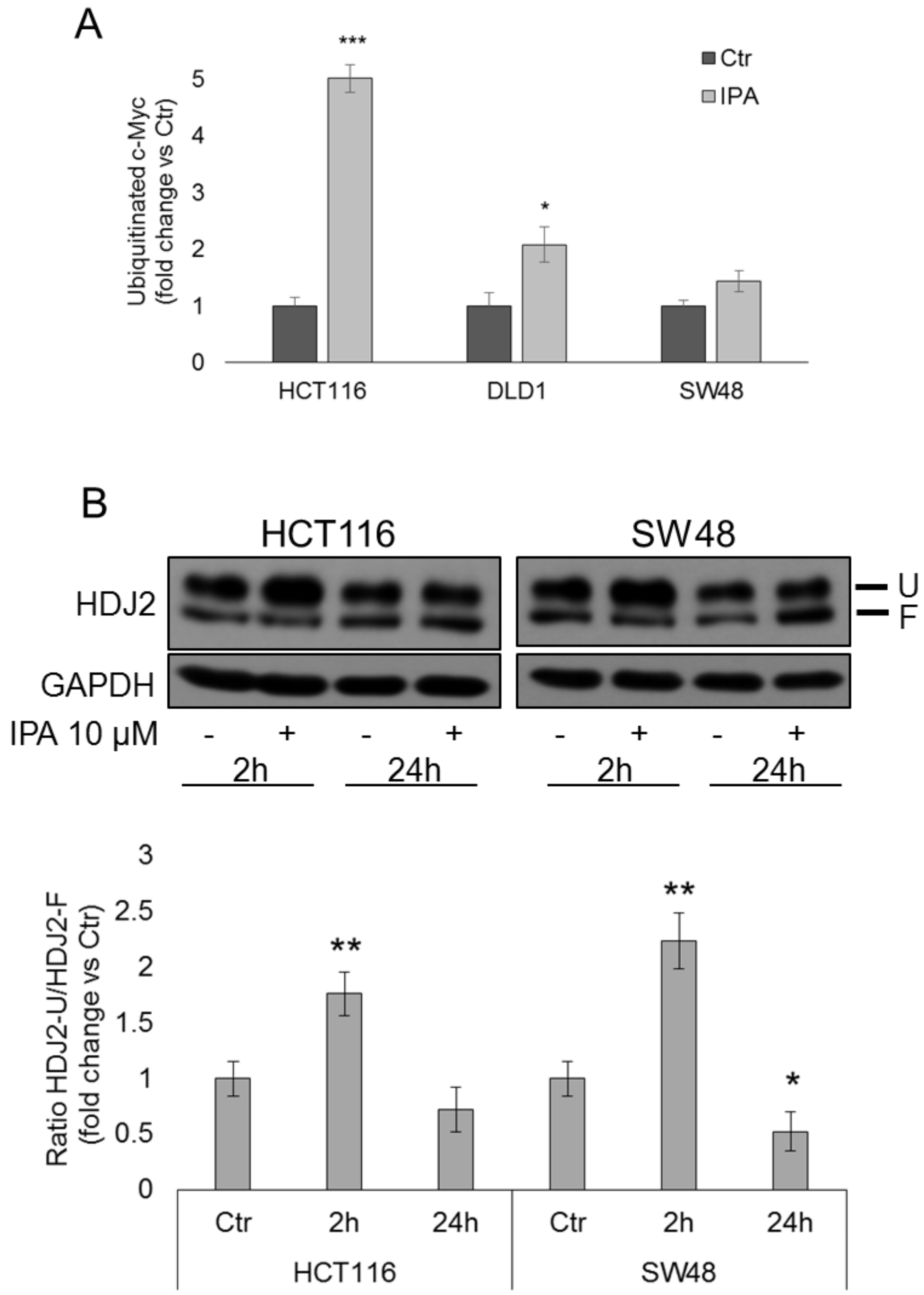

Figure S2. (**A**) Densitometry analysis of Ubiquitinated c-Myc from eluted fraction containing ubiquitinated protein, expressed. (**B**) Representative western blot of mobility-shifted Farnesylated (F) and Unfarnesylated (U) HDJ2 on SDS-PAGE gel, in HCT116 and SW48 cells treated with IPA. The histogram represents densitometry analysis of Unfarnesylated HDJ2 expressed as fold change vs. Farnesylated HDJ2 and normalized versus GAPDH. Data are expressed as mean ± SD of three independent experiments (* $p < 0.05$, ** $p < 0.01$, *** $p < 0.005$ vs. control).

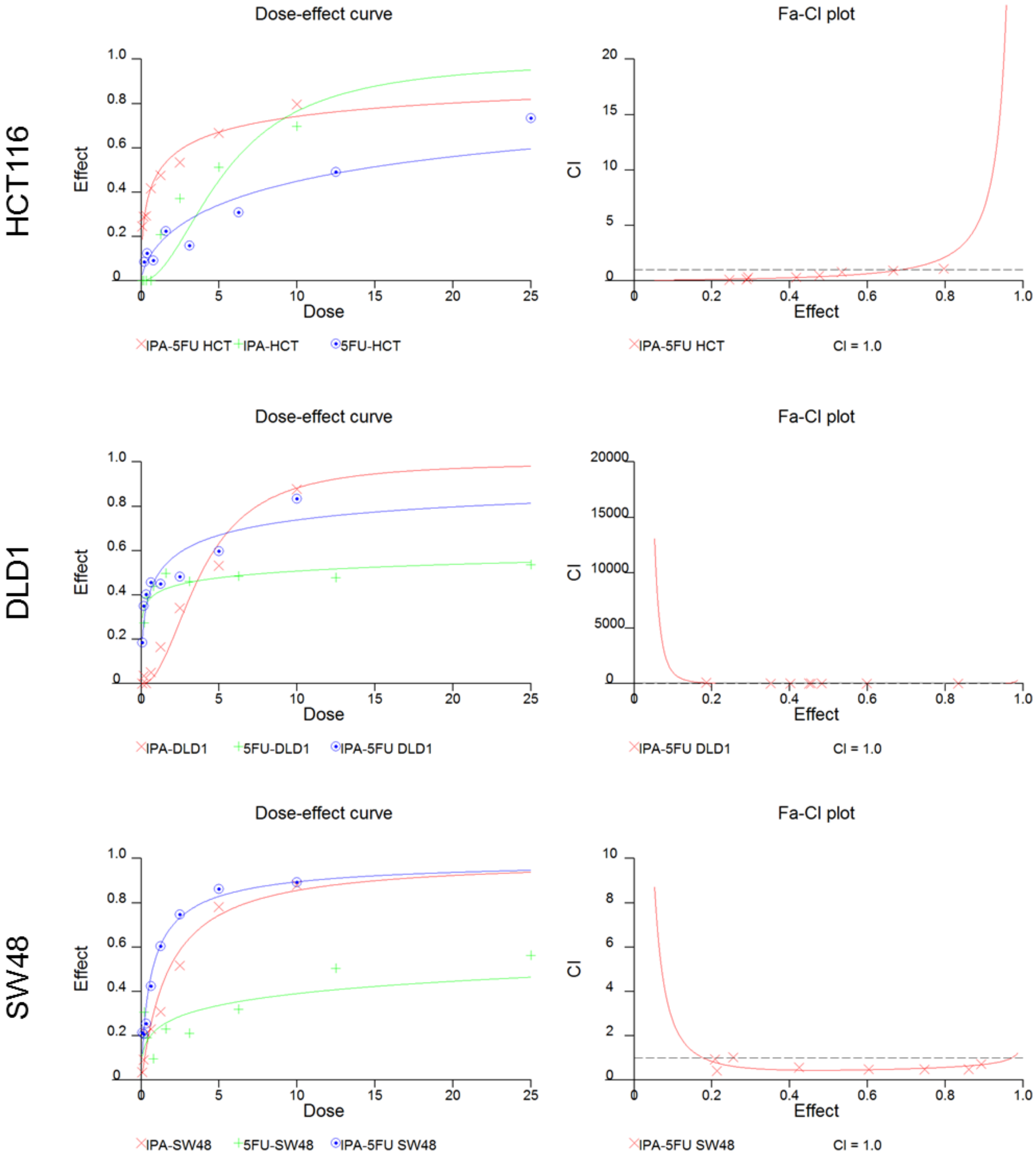

Figure S3. Drug combination analysis. The dose-effect curve was computed using the CalcuSyn software and it showed the dose of the drug vs. the fraction of the cells affected/killed by IPA and 5FU used alone or in combination. Combination Index plot (Fa-CI plot) performed by CalcuSyn software from the affected fraction (Fa) obtained at each dose of the drugs used in combination, at indicated constant molar ratio. A representative experiment of 5 is reported.

Table S1. Molecular interactions of IPA. Putative IPA-interacting proteins emerged from chemical-proteomics experiments. Proteins identified in the cytosolic and in the nuclear fraction are reported separately.

		CYTOSOLIC FRACTION	
**Swiss-Prot CODE**	**Score ***	**Protein**	**Peptides ****
H2B1B_HUMAN	745	Histone H2B type 1-B	5
ENOA_HUMAN	286	Alpha-enolase	3
		NUCLEAR FRACTION	
**Swiss-Prot CODE**	**Score ***	**Protein**	**Peptides ****
H2B1B_HUMAN	916	Histone H2B type 1-B	6
H12_HUMAN	877	Histone H1.2	9
NPM_HUMAN	330	Nucleoplasmin	3
HNRPR_HUMAN	328	Heterogeneous nuclear ribonucleoprotein R	3
THOC4_HUMAN	177	THO complex subunit 4	2

* Average score value achieved in three different chemical-proteomics experiments; ** Number of unique peptide sequences detected in a single experiment. Reported values are the average of the unique sequences detected in three different chemical-proteomics experiments.

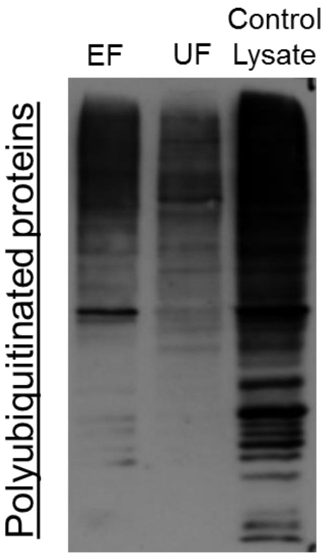

Figure S4. Efficiency of ubiquitinated protein enrichment. Representative western blot of polyubiquitinated proteins in Eluted Fraction (EF) from high-binding affinity matrix, Unbound Fraction (UF) and whole cell lysate used as starting material.

Figure 1 E

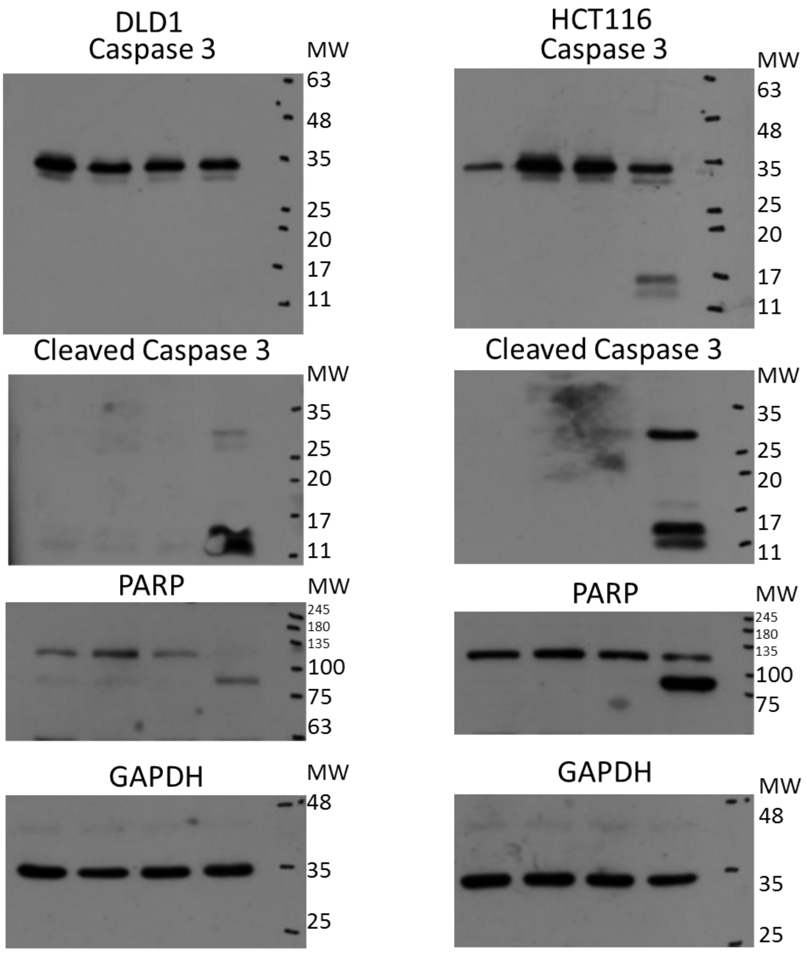

Figure 2A

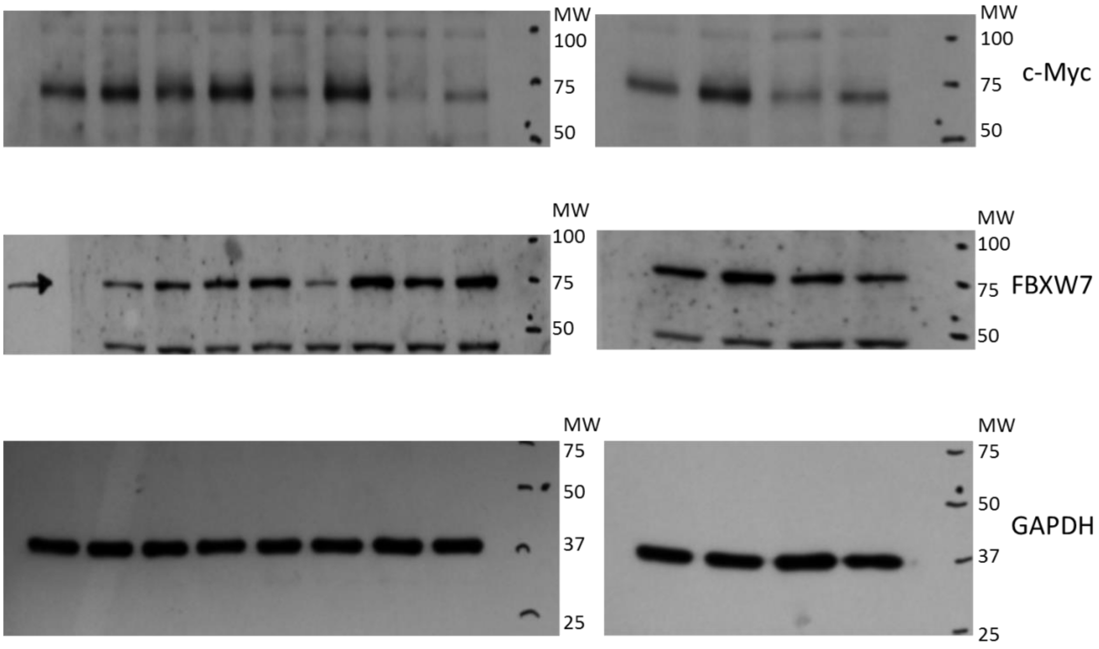

Figure 2B

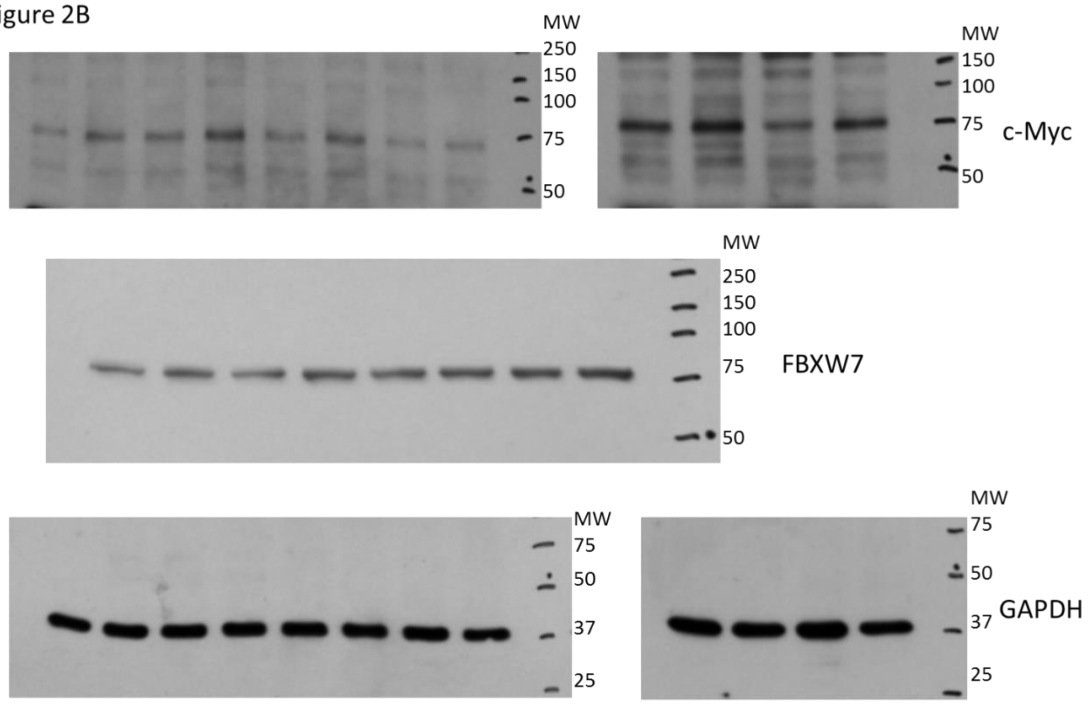

Figure 2C

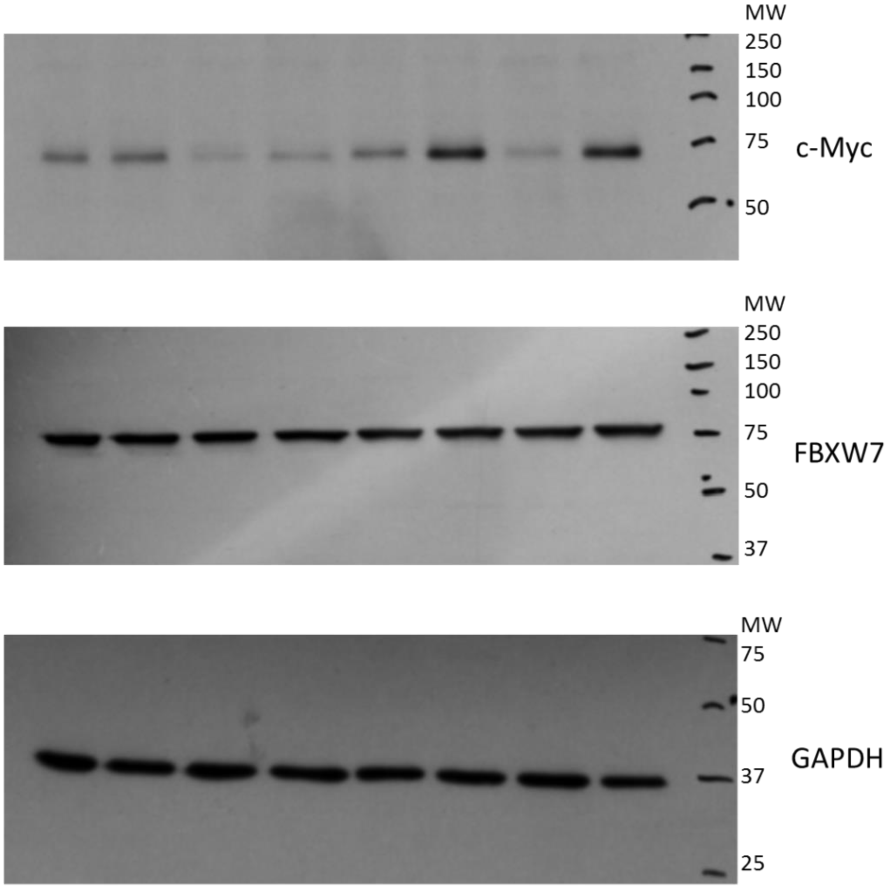

Figure 3B

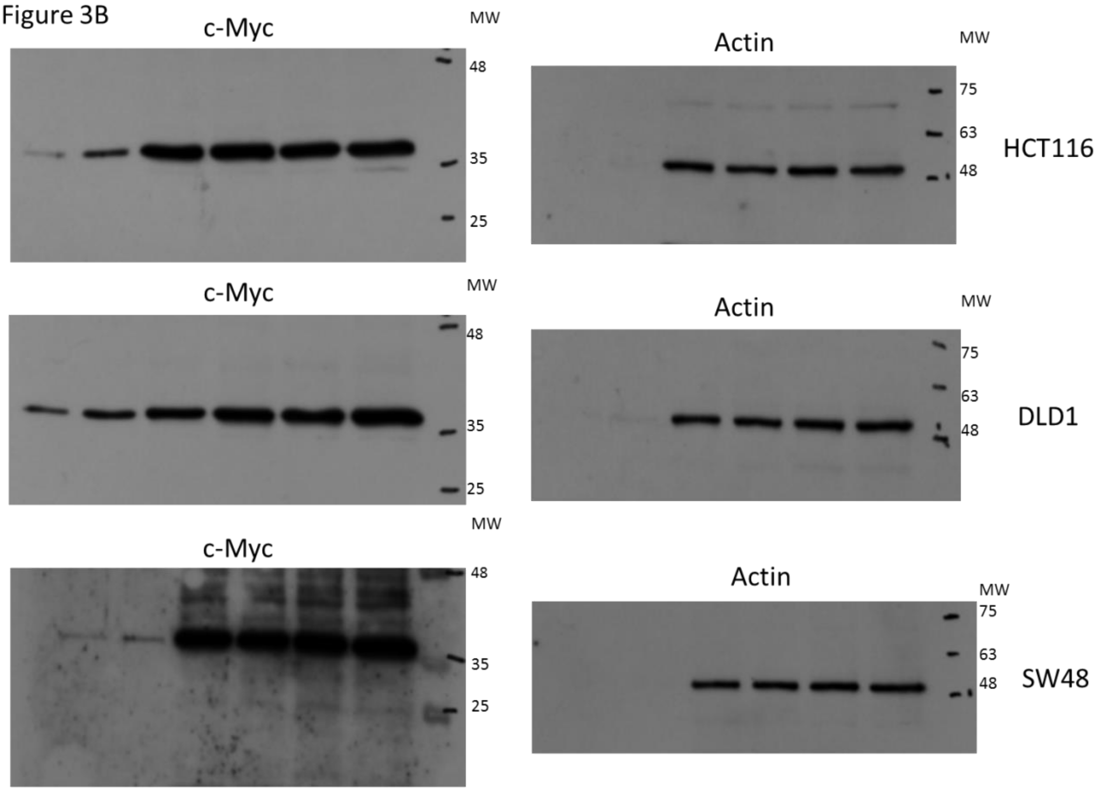

Figure 3C

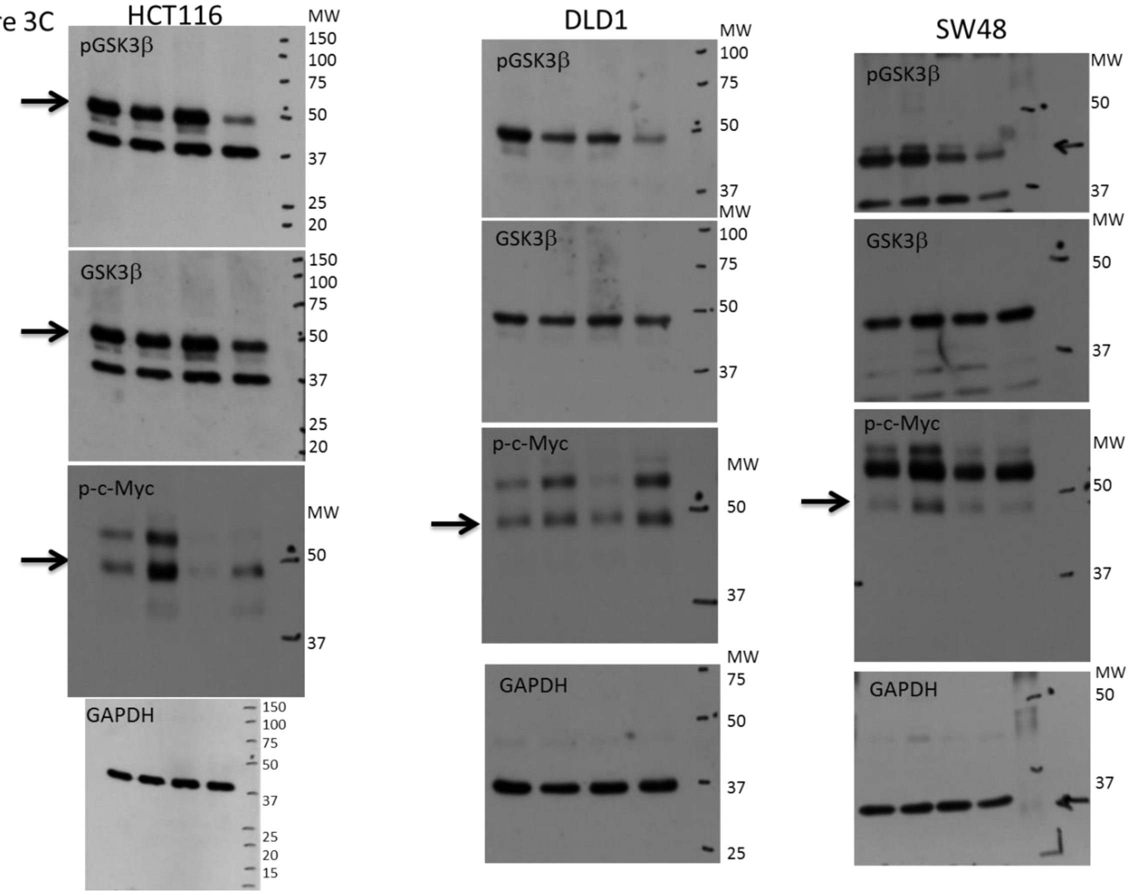

Figure 3D

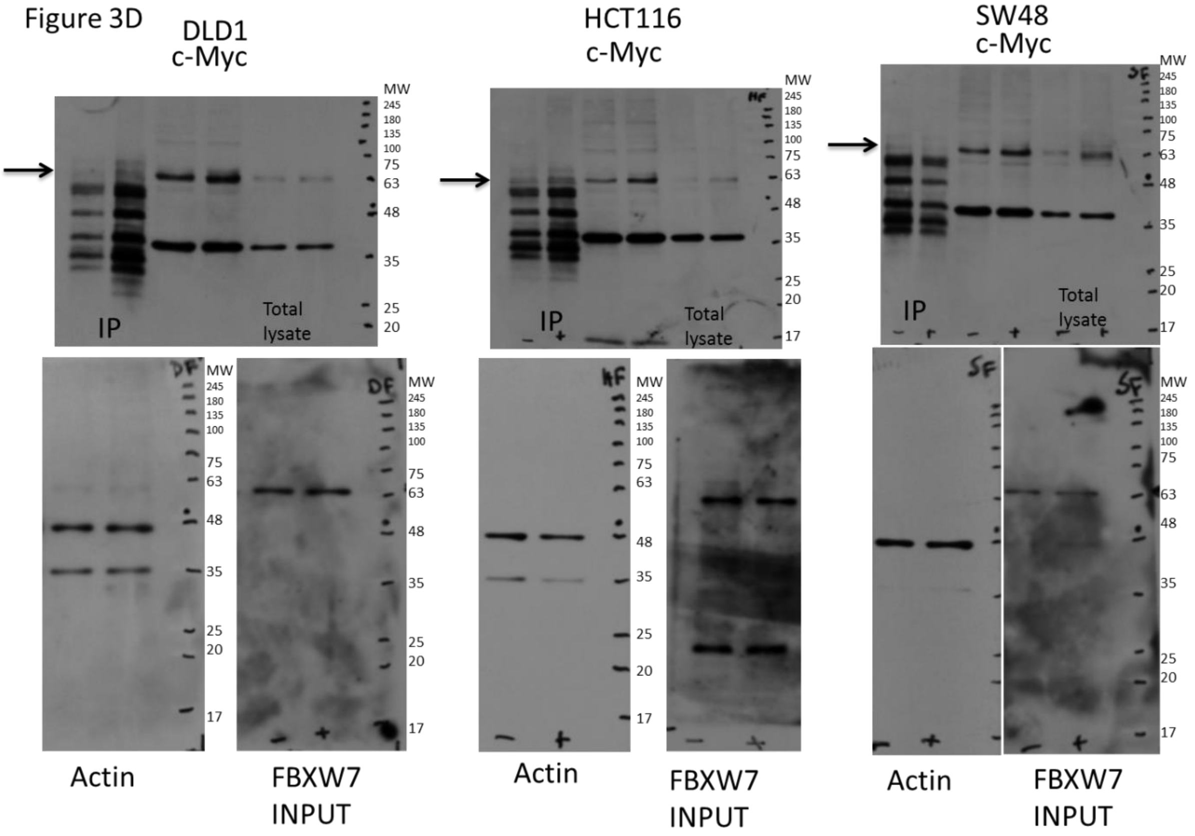

Figure 4A

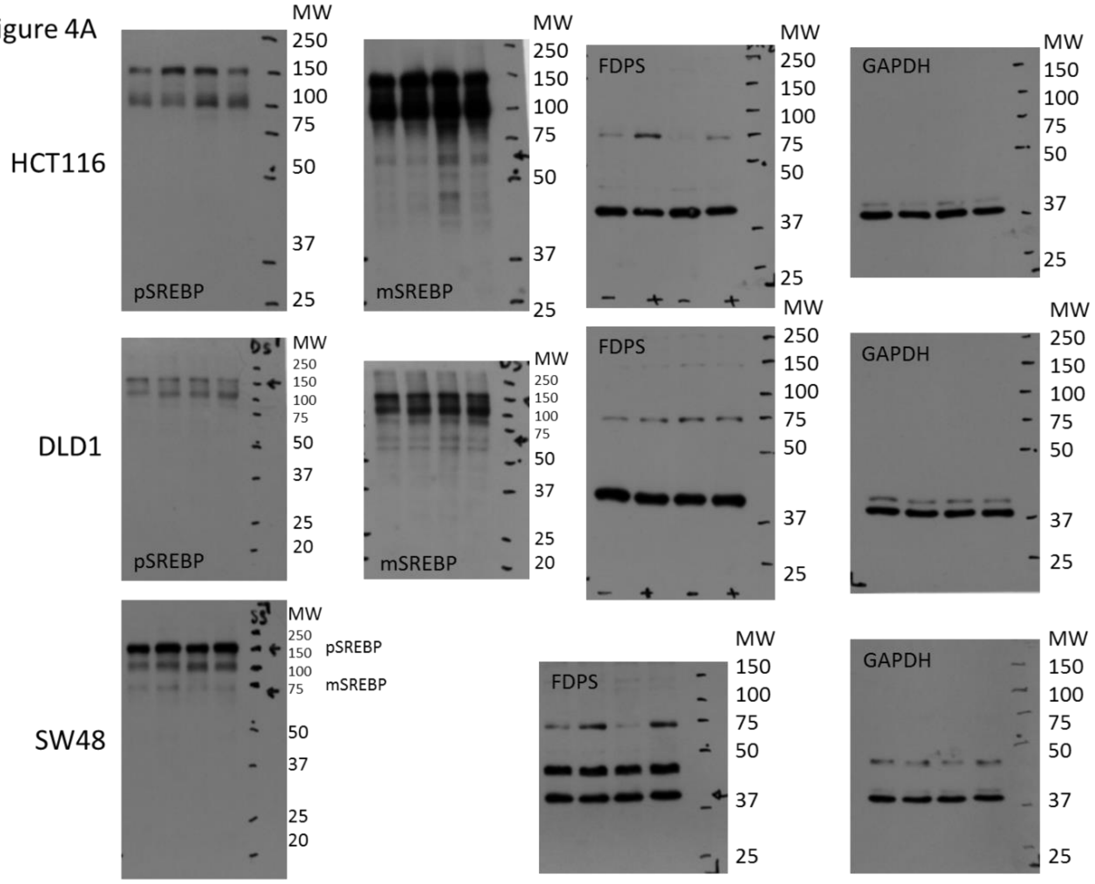

Figure 4C

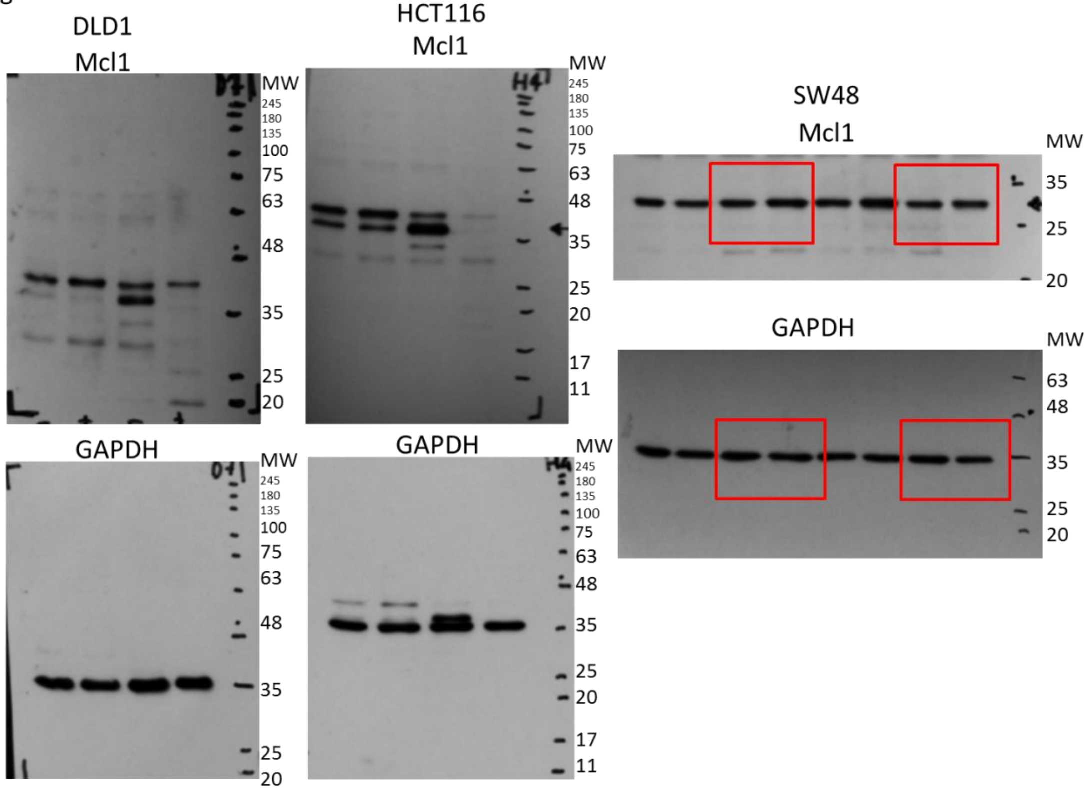

Figure 5A

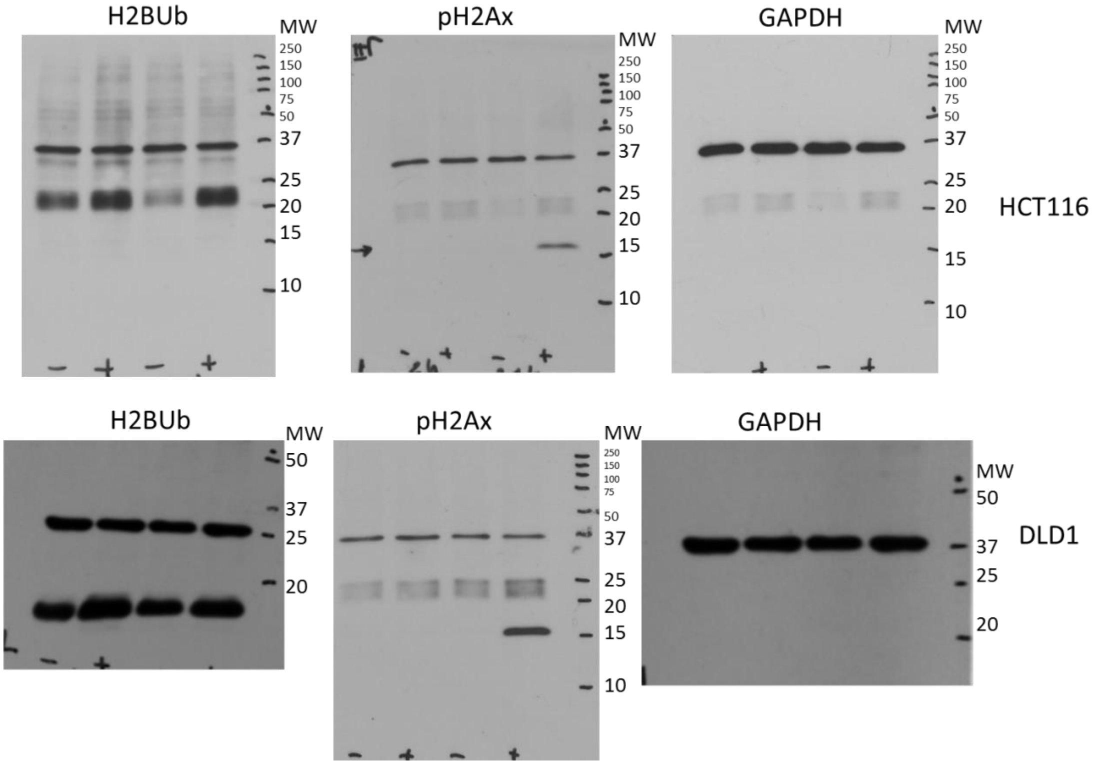

Figure 5B

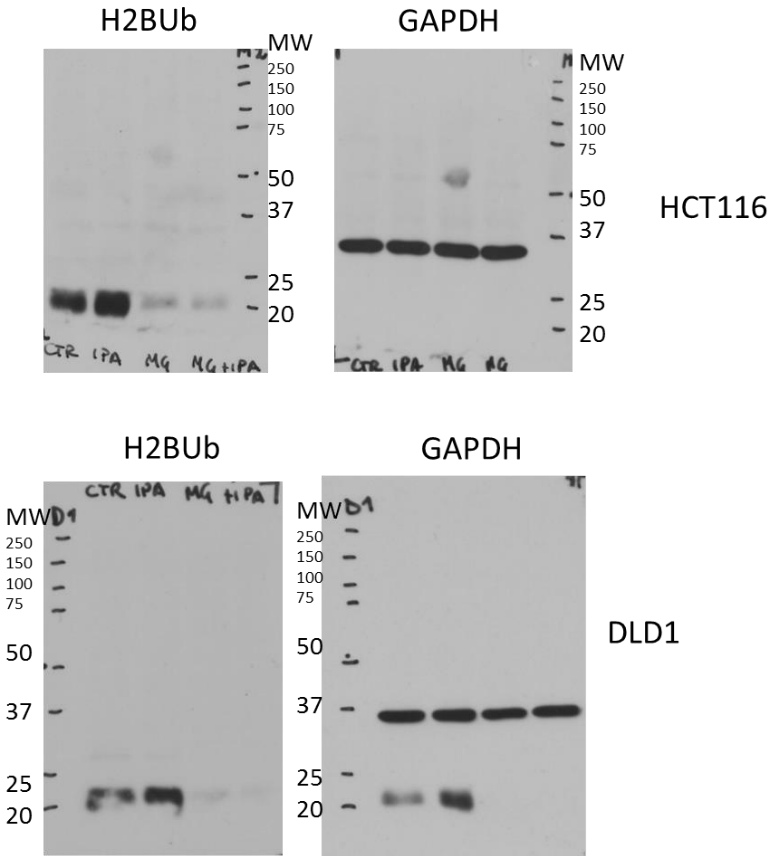

Figure 5C

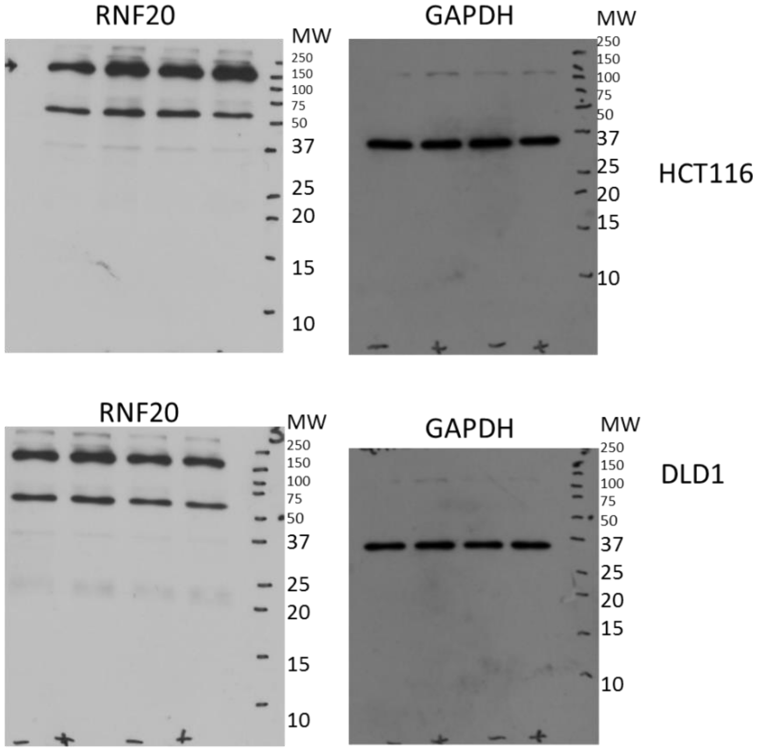

Figure 6B

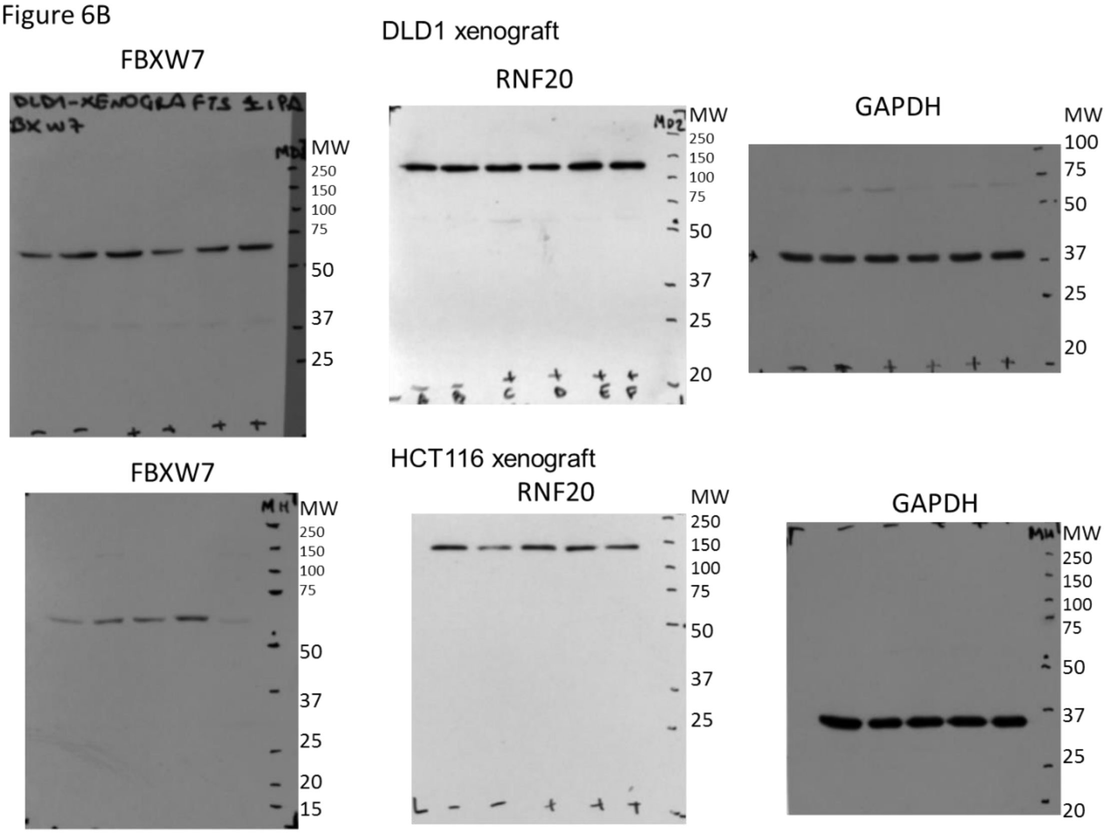

Figure 6C

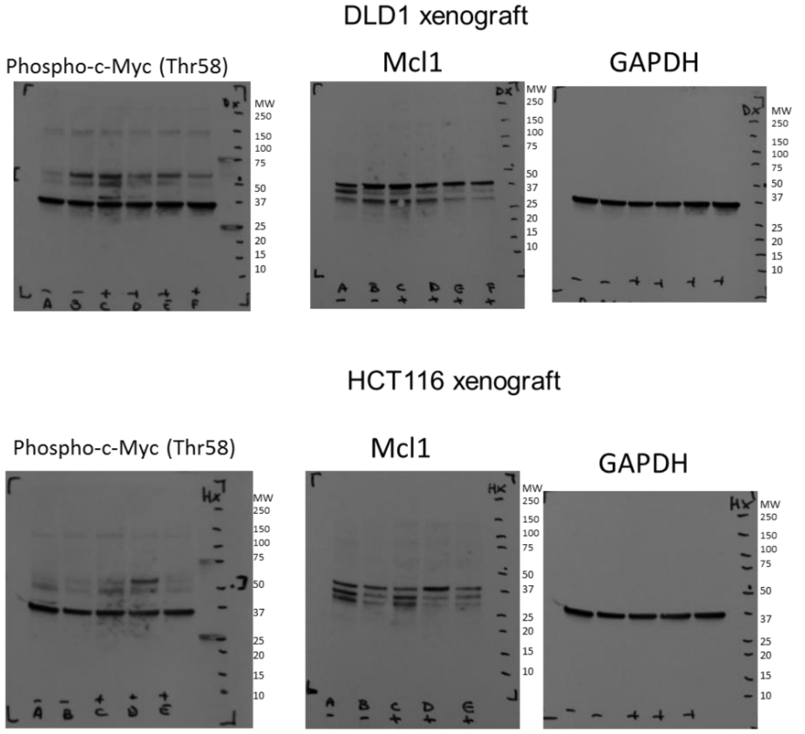

Figure S1

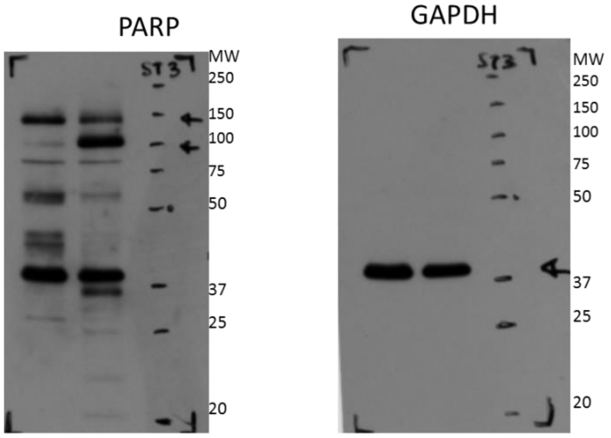

Figure S2

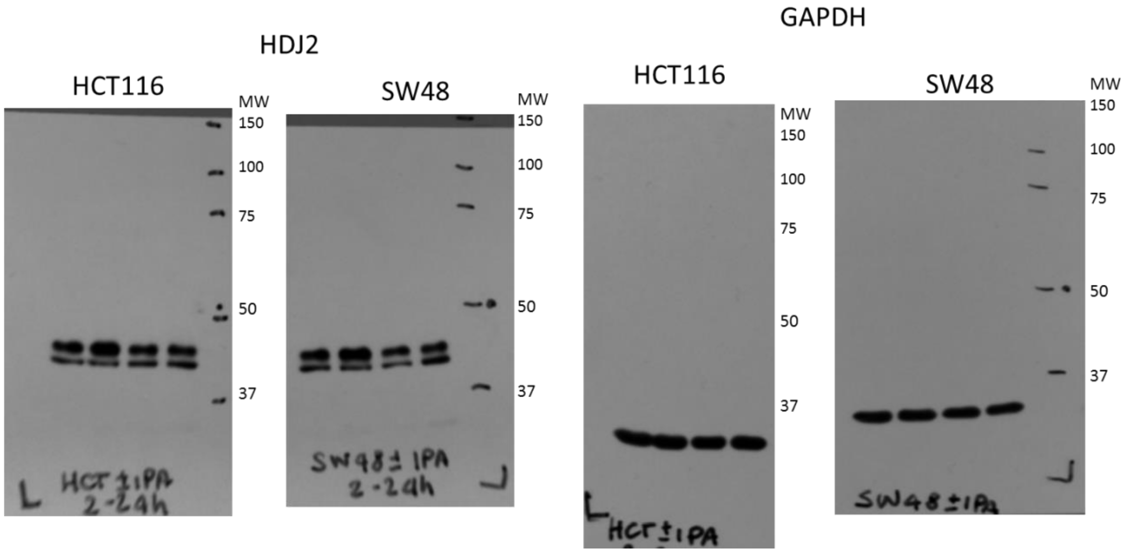

Figure S4

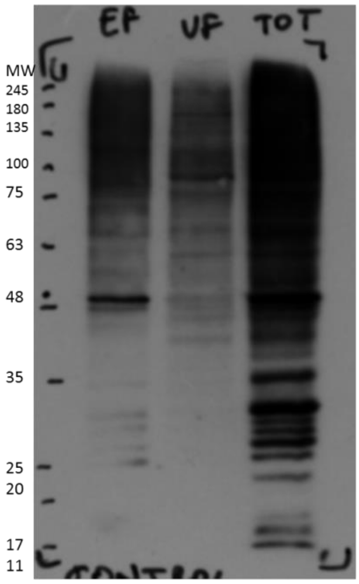

Figure S5. Whole western blots. Uncropped blots corresponding to the relative figures in the main text and showing molecular weight markers.
